# *ORAI1* mutation with mixed loss- and gain-of-function properties causes immunodeficiency and HLH

**DOI:** 10.70962/jhi.20250097

**Published:** 2025-10-30

**Authors:** Lucile Noyer, Priscilla S.-W. Yeung, Sascha Kahlfuss, Catherine Li Lai, Maxwell McDermott, Devisha Patel, Jun Yang, Yin-Hu Wang, Li Zhong, Peter Hsu, Murali Prakriya, Stefan Feske

**Affiliations:** 1 Department of Pathology, New York University Grossman School of Medicine, New York, NY, USA; 2 Department of Pharmacology, Northwestern University Feinberg School of Medicine, Chicago, IL, USA; 3 Department of Allergy and Immunology, The Children’s Hospital at Westmead, Sydney, Australia

## Abstract

Loss-of-function mutations of ORAI1 suppress store-operated Ca^2+^ entry (SOCE) and cause an immunodeficiency disorder called Ca^2+^ release–activated Ca^2+^ channelopathy. Here, we report an infant patient who is compound-heterozygous for p.His134Pro and p.Leu194Pro mutations in ORAI1 and whose T cells have strongly reduced SOCE. Whereas the p.Leu194Pro mutant ORAI1 protein is not expressed at the plasma membrane, the p.His134Pro mutation results in a constitutively open channel that is unresponsive to activation by stromal interaction molecule 1. The patient suffered from a severe form of combined immunodeficiency, hemophagocytic lymphohistiocytosis, and fatal chronic cytomegalovirus infection. His immunodeficiency was characterized by an altered composition of T and NK cell compartments, impaired stimulation-induced cytokine production and signs of CD4^+^ T cell and NK cell activation, but attenuated CD8^+^ T effector memory cell function. Our findings demonstrate that small constitutive SOCE through a mutant ORAI1 channel is not sufficient to provide immunity to viral infection.

## Teaser

A novel mutation in ORAI1 results in constitutive CRAC channel activation while abolishing stimulation-induced channel opening. The mutation is associated with severe immune dysregulation, altered T and NK cell phenotypes and function, and attenuated CD8^+^ T effector memory cell function.

## Graphical Abstract

**Figure:**
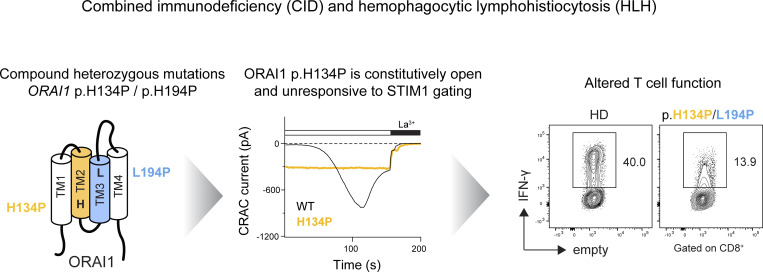

## Introduction

Ca^2+^ signaling is critical for the function of almost all cell types including lymphoid and myeloid immune cells. Ca^2+^ regulates a multitude of cellular processes and immune effector functions such as gene expression, cytokine and chemokine production, metabolic adaptation, proliferation, migration, and phagocytosis (1, 2). Ca^2+^ signals are generated by the release of Ca^2+^ ions from intracellular stores such as the endoplasmic reticulum (ER) and the influx of Ca^2+^ from the extracellular space through specialized Ca^2+^ channels in the plasma membrane (PM). In cells of the immune system, PM Ca^2+^ channels include ATP-activated purinergic receptors such as P2X4 and P2X7, and several homologs of the transient receptor potential channel family including TRPM2 in phagocytes, and TRPM7 in macrophages, T cells, and B cells (3, 4). The most ubiquitous Ca^2+^ channel in immune cells is the Ca^2+^ release–activated Ca^2+^ (CRAC) channel, which mediates a form of Ca^2+^ influx called store-operated Ca^2+^ entry (SOCE). SOCE is induced upon binding of antigen receptors such as the T cell receptor (TCR), B cell receptor, or Fc receptors, and engagement of G protein–coupled receptors including chemokine receptors (4, 5).

Immunoreceptor binding results in the activation of phospholipase C and the production of inositol 1,4,5-trisphosphate (IP_3_), which promotes the opening of IP_3_ receptor channels and Ca^2+^ release from the ER. The subsequent drop in ER Ca^2+^ levels results in the dissociation of Ca^2+^ bound to stromal interaction molecule 1 (STIM1) in the ER membrane, and conformational changes in STIM1 allowing it to relocate to ER regions in close proximity to the PM. There, STIM1 binds to ORAI1, the pore-forming subunit of the CRAC channel, triggering a series of conformational changes in ORAI1 that lead to CRAC channel opening and SOCE (6). ORAI1 is a tetraspanning PM protein, which assembles as a hexameric protein complex to form the CRAC channel (7). The first transmembrane (TM) domains of each ORAI1 subunit line the pore of the channel and form the Ca^2+^ conduction pathway including its selectivity filter. The other TM domains form concentric rings around the pore and are involved in channel gating (6).

SOCE is essential for the function of many immune cells including T and B cells, natural killer (NK) cells, neutrophils, mast cells, and macrophages (3, 8). The function of ORAI1 and STIM1 in immune cells has been studied extensively using knockout mice where it contributes to immunity to infection, antitumor immunity, and allergic responses (1). Disruption of SOCE in humans leads to severely impaired immune responses. Most notably, biallelic loss-of-function (LOF) mutations of *STIM1* and *ORAI1* in humans strongly reduce Ca^2+^ influx and, thus, impair lymphocyte and myeloid cell functions. The disease phenotype associated with LOF mutations in *ORAI1* or *STIM1* genes is called CRAC channelopathy (9). It consists of combined immunodeficiency (CID) with immune dysregulation, especially in the T cell compartment, and several extra-immunological manifestations such as muscular hypotonia and ectodermal dysplasia including defects in dental enamel formation and anhidrosis. Gain-of-function (GOF) mutations in ORAI1 and STIM1 that result in constitutively active CRAC channels, in contrast, are associated with tubular aggregate myopathy (TAM) or Stormorken syndrome (10).

Several patients with LOF mutations in *ORAI1* have been reported. They suffer from severe immunodeficiency during infancy that is associated with an increased susceptibility to a wide range of pathogens, including viruses such as herpes viruses, bacteria such as *Streptococcus pneumoniae*, atypical mycobacteria, and fungi such as *Candida albicans* (9, 11, 12, 13, 14). *ORAI1* LOF mutations are commonly located in one of the four TM domains and abolish either ORAI1 protein expression or channel function. The first *ORAI1* mutation reported is a missense mutation substituting arginine 91 with tryptophan (p.R91W), which blocks the channel pore and suppresses its Ca^2+^ conductance (15, 16). Here, we report a patient who is compound-heterozygous for two mutations in *ORAI1*, p.L194P, and p.H134P. Whereas the L194P mutation interferes with ORAI1 protein expression at the cell surface, the H134P mutation results in a partially constitutively open CRAC channel that cannot be activated by STIM1-mediated gating. Stimulation-induced SOCE is strongly reduced in the patient’s lymphocytes associated with severe immunodeficiency and hemophagocytic lymphohistiocytosis (HLH) that are characterized by a complex immune dysregulation with features of T and NK cell activation and dysfunction.

## Results

### Immune dysregulation in a patient with compound heterozygous mutations in the *ORAI1* gene

**Figure S1.**
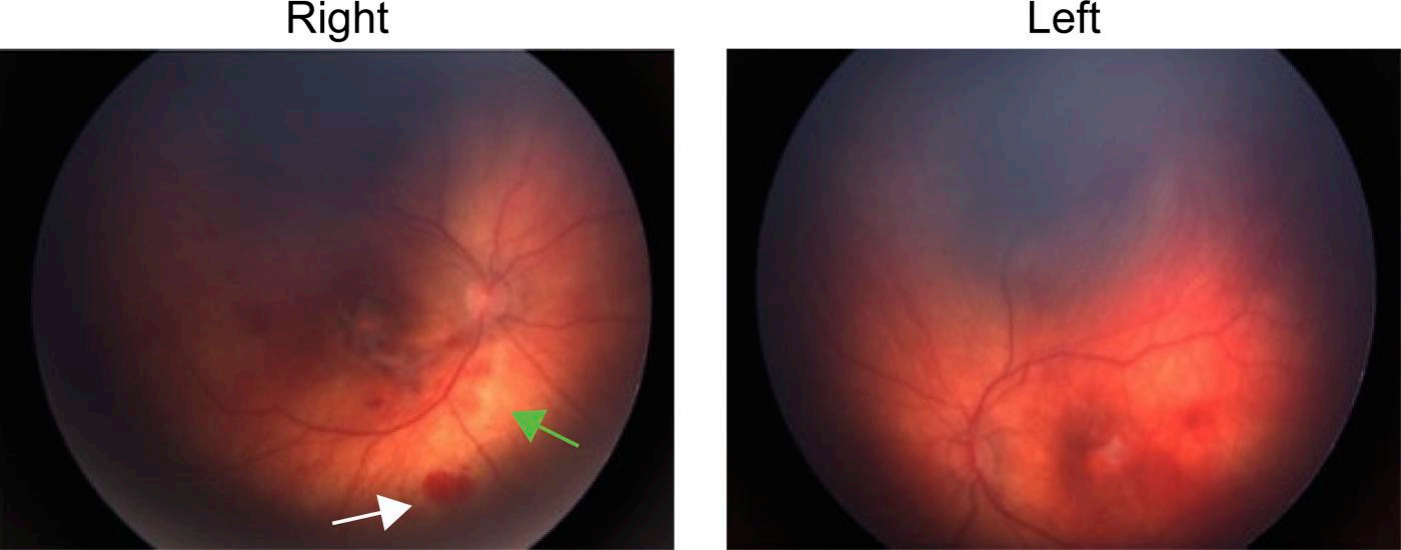
**CMV retinitis in the ORAI1 mutant patient.** Fundoscopy pictures of the ORAI1 mutant patient consistent with CMV retinitis. The green arrow indicates a granular yellow-white lesion along the vasculature; the white arrow shows hemorrhages.

**Figure 1.**
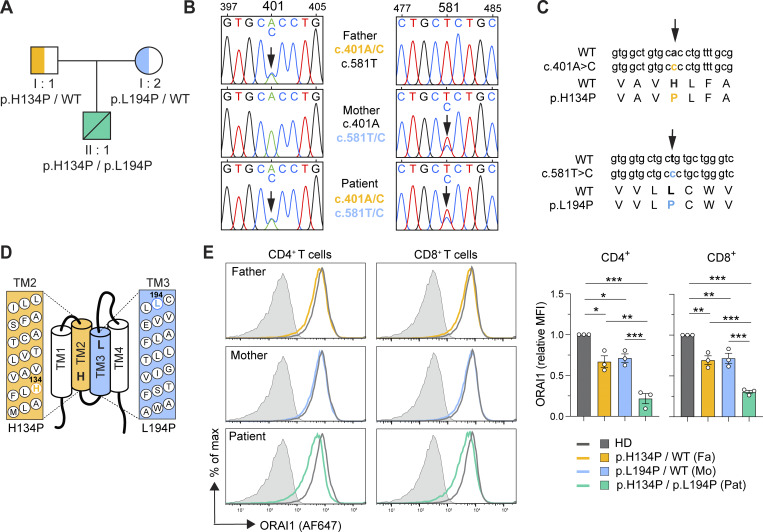
**Compound heterozygosity for two *ORAI1* mutations reduces protein expression. (A)** Pedigree of patient with *ORAI1* mutation and his unrelated parents. **(B)** Sanger sequencing of *ORAI1* gene of the patient, father, and mother. Numbers refer to nucleotide positions in the *ORAI1* coding sequence (mRNA). **(C)** Location of the patient’s missense mutations compared with reference sequences of *ORAI1* mRNA (NM_032790.4) and ORAI1 protein (NP_116179.2). **(D)** Locations of ORAI1 missense mutations overlaid on the membrane topology of the ORAI1 protein. **(E)** Flow cytometry analysis of cell surface ORAI1 protein expression in CD4^+^ and CD8^+^ T cells from the patient (Pat), his father (Fa), mother (Mo), and an HD control. Nonpermeabilized T cells were incubated with an Alexa Fluor 647–conjugated anti-human ORAI1 mAb (2C1.1) recognizing an epitope in the second extracellular loop of ORAI1 (17, 18). Representative histogram plots (left) and quantification of the mean fluorescence intensity (MFI) of ORAI1, normalized to the MFI of HD (right). Data are from three independent experiments. Statistical significance was calculated using ordinary one-way ANOVA. For CD4^+^ T cells: P = 0.0105; 0.0226; <0.0001; 0.0016; 0.0008. For CD8^+^ T cells: P = 0.0042; 0.0065; <0.0001; 0.0009; 0.0006. *P < 0.05, **P < 0.01, ***P < 0.001.

We investigated a 4-mo-old infant who presented with pneumococcal sepsis and persistent cytomegalovirus (CMV) viremia complicated by CMV retinitis (Fig. S1), pneumonitis, and HLH that was characterized by hepatosplenomegaly, anemia, thrombocytopenia, hyperferritinemia, and hypertriglyceridemia. Further investigation revealed features of CID, generalized hypotonia with gross motor developmental delay, hypotrichosis, and probable anhidrosis, consistent with the phenotype reported in patients with CRAC channelopathy (9). Detailed clinical information is provided in the case report in Materials and methods and Table 1. Whole-exome sequencing revealed that the patient is compound-heterozygous for two distinct mutations in the *ORAI1* gene (Fig. 1, A–D). One of the mutations is a c.581T>C missense mutation resulting in a predicted leucine-to-proline amino acid substitution at position 194 of the ORAI1 protein located in TM3 (p.L194P, Fig. 1, A–D). The patient’s mother, but not the father, is heterozygous for the same c.581T>C mutation indicating maternal transmission of this allele. The p.L194P mutation has previously been reported in two unrelated patients with CRAC channelopathy and impaired SOCE (12, 13, 14). The second variant identified in the patient is a c.401A>C missense mutation that results in a predicted histidine-to-proline substitution at position 134 of the ORAI1 protein located in transmembrane segment 2 (TM2) (p.H134P, Fig. 1, A–D). The patient’s father, but not his mother, is heterozygous for the same c.401A>C mutation indicating paternal transmission of this allele. A missense mutation at this residue has not been reported before, and its significance was therefore unknown. Of note, the mother and father were both healthy and did not show symptoms of CRAC channelopathy, indicating that haploinsufficiency for ORAI1 is not detrimental, consistent with previous findings in heterozygous carriers of autosomal recessive LOF mutations in *ORAI1* (12, 13, 15). These findings moreover suggested that both mutations are pathogenic since the p.H134P mutation fails to rescue the known detrimental effects of the p.L194P mutation, and vice versa.

**Table 1.** Summary of genetic, clinical, and laboratory findings of the patient (*ORAI1* p.H134P/L194P)

Clinical features	
Age at presentation	4 mo
Age at death	13 mo
Mutation	*ORAI1* c.401A>C (p.H134P); c.581T>C (p.L194P)
Type of mutation	GOF/LOF (p.H134P); LOF (p.L194P)
Mode of inheritance	AR
Infections	Pneumococcal sepsis, CMV infection (viremia, retinitis, pneumonitis)
Muscular hypotonia	Yes
Anhidrosis	Probable
Enamel development defect	N/A
Hypotrichosis	Yes
Age at HSCT	10 mo
Donor	10/10 matched unrelated donor
Number of transplants	3
Graft used	Sorted CD34^+^ stem cells; donor CMV-specific cytotoxic T cells
Conditioning used	Treosulfan, fludarabine
HSCT complications	Graft failure, severe graft-vs-host disease, persistent CMV infection respiratory failure, severe neurological inflammation

​	Patient	Reference range
*CBC* ^a^		
White cell count (10^9^/Liter)	6.7	5.0–17.0
Lymphocytes (10^9^/Liter)	4.0	2.0–13
Monocytes (10^9^/Liter)	0.2	0.2–1.2
Neutrophil (10^9^/Liter)	2.0	0.8–8
Eosinophil (10^9^/Liter)	0.5	0-1.1
*Immunological results* ^a^		
CD4 T cells (×10^9^/Liter)	2.44	1.5–5.0
CD8 T cells (×10^9^/Liter)	**0.40**	0.50–1.60
NK cells (×10^9^/Liter)	0.12	0.1–1.3
B cells (×10^9^/Liter)	1.00	0.60–3.00
IgM (g/Liter)	**2.06**	0.24–0.95
IgG (g/Liter)	**19**	1.68–5.76
IgA (g/Liter)	**3.7**	0.05–0.74
NK cell function (^51^Cr release assay and degranulation [CD107a expression])	Significantly reduced compared with HD	N/A
Proliferation to PHA stimulation *in vitro*	Significantly reduced compared with HD	N/A
Immune dysregulation	HLH^b^:	​
	Fever	​
	Hepatosplenomegaly	​
	Anemia (Hb 74 g/l)	95-140
	Thrombocytopenia (73 × 10^9^/Liter)	150-600
	Hypofibrinogenemia (0.6 g/Liter)	1.5–6
	Hyperferritinemia (1,293 µg/Liter)	30-300
	Hypertriglyceridemia (3.2 mmol/Liter)	0.5–1.4
	High LDH (2116 U/Liter)	313-618

Abbreviations: AR, autosomal recessive; PBMC, peripheral blood mononuclear cell; CMV, cytomegalovirus; Ig, immunoglobulin; HSCT, hematopoietic stem cell transplantation; GvHD, graft-versus-host disease; HD, healthy donor; PHA, phytohemagglutinin. The reference range is adjusted for age. Bold values are outside the reference range.

a Age of analysis: 5 mo.

b Age at analysis: 4 mo.

### Compound heterozygosity for ORAI1 mutations decreases channel expression at the cell surface

**Figure 2.**
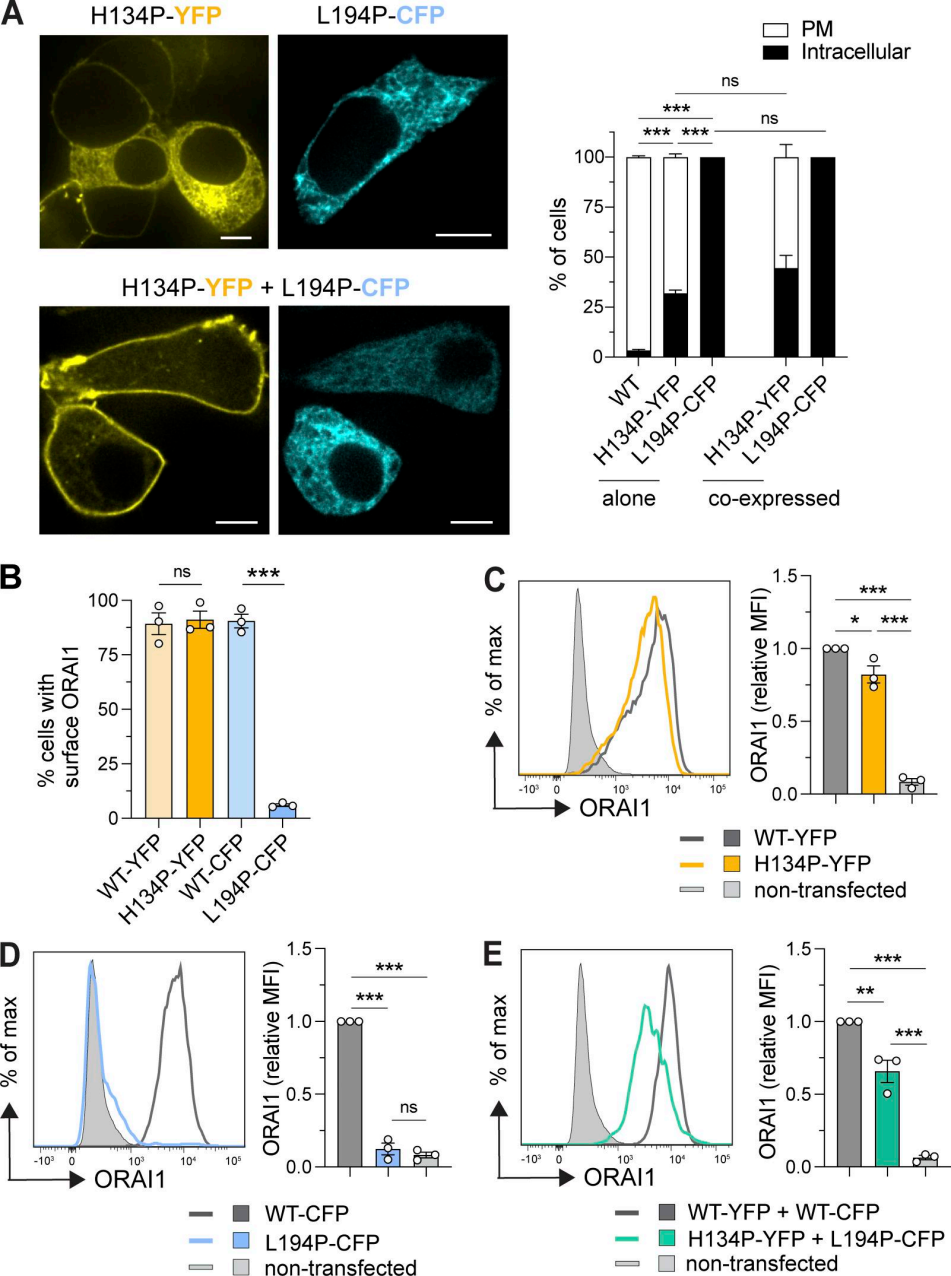
**Reduced expression of the ORAI1 mutant protein at the cell surface. (A)** Cellular localization of ORAI1 in HEK293 cells overexpressing either ORAI1-WT-YFP, ORAI1-H134P-YFP, or ORAI1-L194P-CFP. Representative confocal microscopy images (left), and quantification of frequencies of cells having PM or intracellular ORAI1 expression patterns (right). Scale bars are 5 µm. Data are from 200 to 400 cells per condition and 5 independent experiments. Cells with low expression (<5× background intensity) were excluded from the analysis. P ≤ 0.0001; <0.0001; <0.0001; 0.0777; >0.9999. **(B–E)** Flow cytometry analysis of cell surface ORAI1 protein expression in HEK293 cells. Nonpermeabilized cells were stained with a mouse anti-human ORAI1 (29A2) mAb that recognizes amino acids 196–234 in the second extracellular loop of hORAI1 (19). **(B)** Cells were transfected with either ORAI1-WT-YFP, ORAI1-H134P-YFP, ORAI1-WT-CFP, or ORAI1-L194P-CFP. Frequency of cells expressing ORAI1 at the cell surface based on the gating strategy shown in Fig. S2 C (P = 0.9233; <0.0001). **(C–E)** Representative flow cytometry plots (left) and quantification of MFI of ORAI1 mutants normalized to the corresponding ORAI1-WT control (right). Data are from three independent experiments. **(C)** Cells were transfected with either ORAI1-WT-YFP, ORAI1-H134P-YFP, or nontransfected cells. P = 0.0299; <0.0001; <0.0001. **(D)** Cells were transfected with either ORAI1-WT-CFP, ORAI1-L194P-CFP, or nontransfected cells. P = <0.0001; <0.0001; 0.5304. **(E)** Cells were cotransfected with either ORAI1-WT-YFP and ORAI1-WT-CFP, ORAI1-H134P-YFP and ORAI1-L194P-CFP, or nontransfected cells. P = 0.0041; <0.0001; 0.0002. Statistical significance was calculated using two-way ANOVA (A) and ordinary one-way ANOVA (B–E). ns, not significant, *P <0.05, **P <0.01, ***P <0.001.

**Figure S2.**
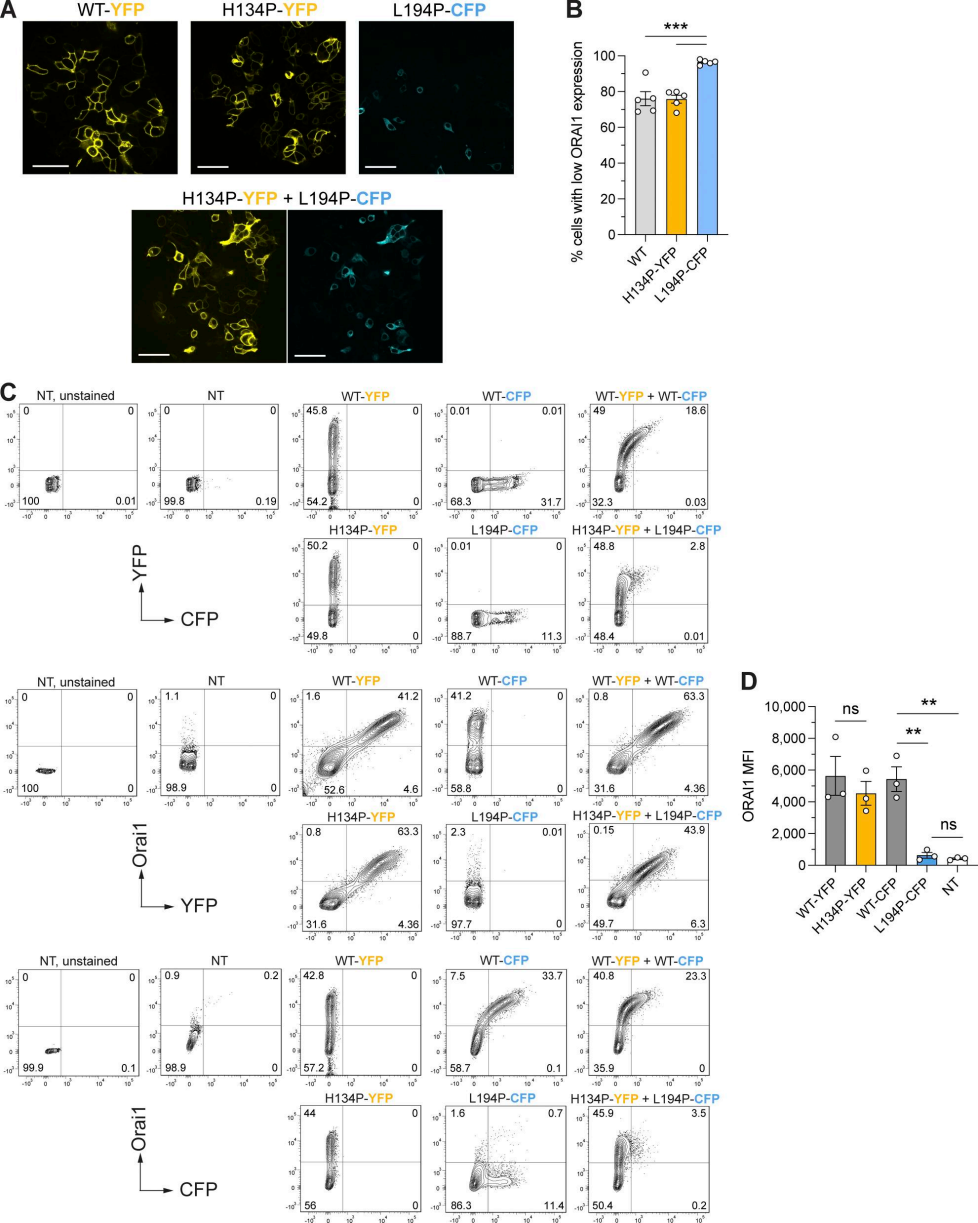
**Reduced ORAI1-L194P expression and retention in the cytosol. (A and B)** Cellular localization of ORAI1 in HEK293 cells overexpressing either ORAI1-WT-YFP, ORAI1-H134P-YFP, or ORAI1-L194P-CFP. **(A)** Representative confocal microscopy images. **(B)** Quantification of frequencies of cells with low ORAI1 expression levels (<5× background intensity). Scale bars are 50 µm. Data are from 200 to 400 cells per condition, from 5 independent experiments. P = 0.0004; 0.0003. **(C)** Flow cytometry analysis of cell surface ORAI1 protein expression in HEK293 cells. Cells were either transfected with ORAI1-WT-YFP, ORAI1-WT-CFP, ORAI1-H134P-YFP, ORAI1-L194P-CFP, or NT. Nonpermeabilized cells were stained with mouse anti-human ORAI1 (2C11) mAb against the second ECD. Representative flow cytometry plots. **(D)** Raw, non-normalized MFI of ORAI1 in all cells per condition. P = 0.821; 0.0066; 0.0049; 0.9995. Statistical significance was calculated using ordinary one-way ANOVA. ns, not significant, **P < 0.01, ***P < 0.001. NT, nontransfected.

To investigate the effects of ORAI1 mutations, we first analyzed the cell surface expression of ORAI1 in CD4^+^ and CD8^+^ T cells isolated from the patient, his parents, and a healthy donor (HD) control, using a monoclonal anti-ORAI1 antibody that recognizes the second extracellular domain (ECD) of human ORAI1 (17). The patient showed a significant reduction of ORAI1 protein levels at the surface of T cells (Fig. 1 E), suggesting that the p.H134P and p.L194P mutations interfere with ORAI1 protein expression or trafficking to the PM. CD4^+^ and CD8^+^ T cells from both the mother and father also showed a decrease of ORAI1 PM levels, which was significant but smaller than that observed in the patient. To gain insights into the mechanism by which each of the two mutations reduced ORAI1 levels at the PM, we expressed wild-type (WT) and mutant ORAI1 proteins fused to cyan fluorescent protein (CFP) or yellow fluorescent protein (YFP) in HEK293 cells followed by analysis of ORAI1 subcellular localization using confocal microscopy. The majority of ORAI1-WT (fused to YFP) was found at the PM with only 5% of cells displaying an intracellular expression pattern (Fig. 2 A), indicating that overexpressed ORAI1 is efficiently transported to the PM and not trapped in the ER. Overexpressed ORAI1-H134P (YFP) was readily detectable, and the frequencies of cells expressing high levels of mutant ORAI1 were comparable to those of ORAI1-WT (Fig. S2, A and B). The majority of ORAI1-H134P (∼70%) localized to the PM, whereas in a minority of cells (∼30%), ORAI1-H134P was found intracellularly (Fig. 2 A). These findings suggest that the p.H134P mutation does not interfere with ORAI1 protein expression as such, but results in a partial defect in ORAI1 localization at the PM. In contrast, the overall levels of ORAI1-L194P (CFP) appeared lower than those of ORAI1-WT or ORAI1-H134P (Fig. 2 A; and Fig. S2, A and B). The residual expressed mutant ORAI1-L194P was found almost entirely intracellularly without robust localization to the PM. Together, these data suggest that the p.L194P mutation interferes with ORAI1 protein expression and its localization to the PM. We hypothesized that in the compound heterozygous patient, the mutant ORAI1-H134P protein might coassemble with ORAI1-L194P and, thus, stabilize ORAI1-L194P expression and facilitate its localization to the PM. We therefore coexpressed both mutants in HEK293 cells. Coexpression did not, however, alter the localization of ORAI1-L194P or ORAI1-H134P compared with their individual expression, suggesting that ORAI1 channels in the PM of the patient’s cells exclusively consist of homomeric ORAI1-H134P channels.

To confirm these findings and directly analyze ORAI1 at the PM of nonpermeabilized cells, we measured ORAI1 cell surface levels in HEK293 cells overexpressing WT and mutant channel proteins by flow cytometry using an antibody against the second ECD of ORAI1. The frequencies of cells expressing either ORAI1-WT or ORAI1-H134P (YFP) at the cell surface were comparable (Fig. 2 B and Fig. S2 C). Cells expressing ORAI1-H134P showed only a modest decrease in surface ORAI1 expression measured by mean fluorescence intensity compared with ORAI1-WT (YFP) (Fig. 2 C; and Fig. S2, C and D). In contrast, the levels of ORAI1-L194P (CFP) protein expression at the cell surface were strongly reduced compared with ORAI1-WT (CFP) and comparable to levels in nontransfected cells (Fig. 2 D; and Fig. S2, C and D). Moreover, only <10% of cells transduced with ORAI1-L194P had detectable protein at the cell surface (Fig. 2 B and Fig. S2 C). We again investigated whether coexpression of both mutants mitigated the expression defect of the ORAI1-L194P mutant. The surface expression of ORAI1 in cells expressing both ORAI1-H134P and ORAI1-L194P remained significantly lower than in cells ectopically expressing ORAI1-WT (Fig. 2 E; and Fig. S2, C and D). Together, our data show that the p.H134P mutation has only moderate effects on total ORAI1 protein levels and those at the PM, whereas the p.L194P mutation strongly reduces both.

### ORAI1-H134P mutation results in constitutive CRAC current that is insensitive to STIM1 gating

**Figure 3.**
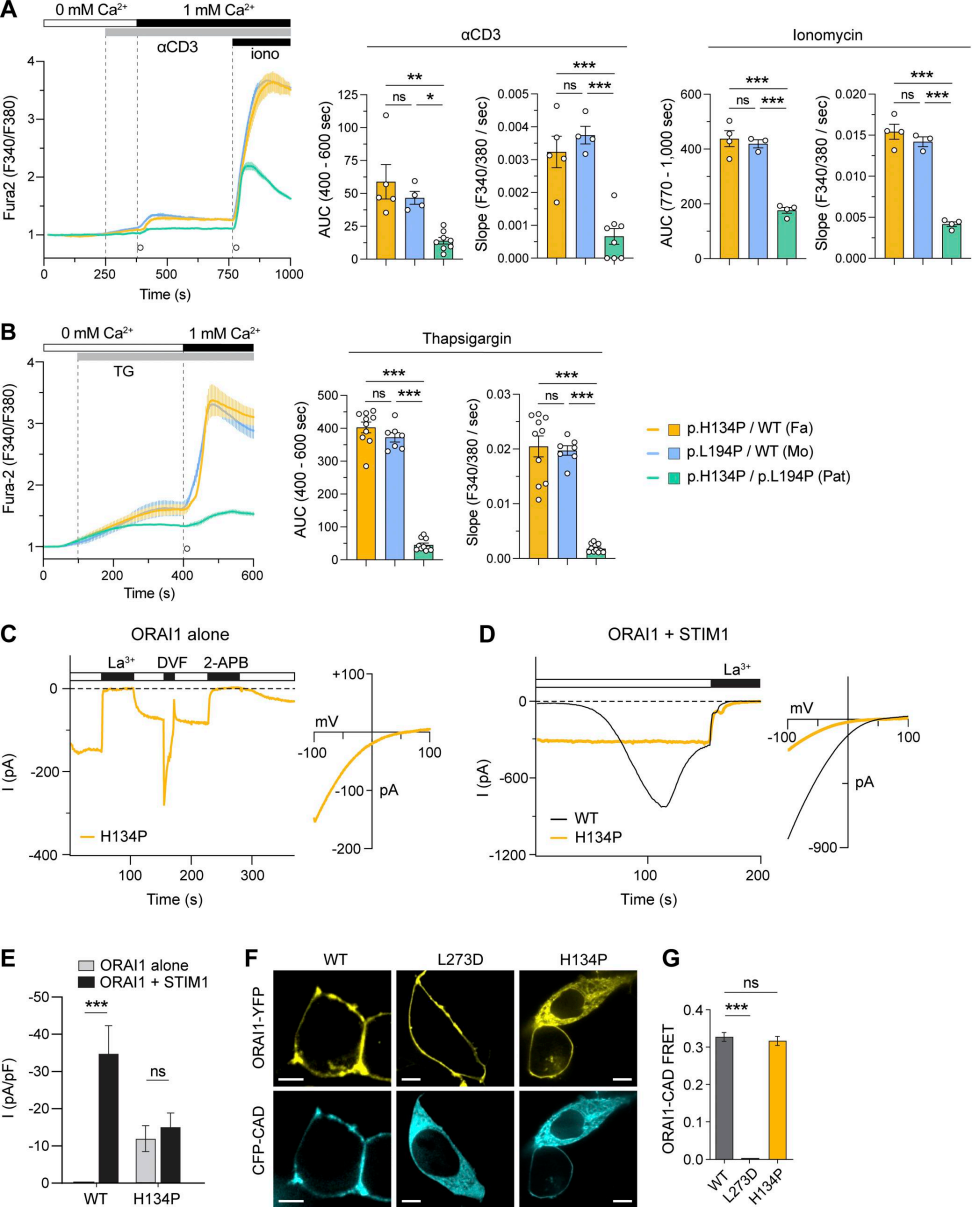
**Decreased SOCE in patient T cells and loss of STIM1 gating in cells expressing ORAI1-H134P mutation. (A and B)** Calcium measurements in in vitro–expanded T cells from the patient (Pat), his father (Fa), and mother (Mo) using Fura-2 AM. Representative time course of Fura-2 fluorescence ratios (340/380 nm) over time (left). Quantified area under the curve (AUC) and slope of Ca^2+^ influx at times indicated by circles (right). Data are from at least four independent experiments. **(A)** Cells were placed in 0 mM Ca^2+^ Ringer’s solution, and SOCE was induced by anti-CD3 cross-linking, followed by the addition of 1 mM Ca^2+^ Ringer’s solution, and stimulation with 1 µM ionomycin (iono). For anti-CD3 AUC: P = 0.6584; 0.0012; 0.0223. For anti-CD3 slope: P = 0.7256; 0.0002; <0.0001. For iono AUC: P = 0.9146; <0.0001; 0.0001. For iono slope: P = 0.5677; <0.0001; <0.0001. **(B)** Cells were placed in 0 mM Ca^2+^ Ringer’s solution, and SOCE was induced by stimulation with 1 µM TG, followed by the addition of 1 mM Ca^2+^. AUC: P = 0.3418; <0.0001; <0.0001. Slope: P = 0.9719; <0.0001; <0.0001. **(C–E)** Whole-cell patch-clamp recordings of HEK293 cells. CRAC currents were activated by passive depletion of ER Ca^2+^ stores with 8 mM BAPTA in the patch pipette and recorded in the whole-cell recording configuration. The plots show the peak whole-cell current at −100 mV (in pA) during steps to −100 mV applied every second following whole-cell break-in. For each condition, the I-V curves are shown on the right. The I-Vs are averages of 6-10 ramps collected between 50 and 60 s (C), 5 and 15 s (H134P ORAI1+STIM1), and 110 and 115 s (WT ORAI1+STIM1) (D). **(C)** Cells were transfected with ORAI1-H134P alone. Time course of current (left) and I-V relationship (right). **(D)** Cells were transfected with STIM1 together with either ORAI1-WT or ORAI1-H134P. **(E)** Current densities normalized to the cell capacitance of ORAI1-WT and ORAI1-H134P with or without STIM1 measured at whole-cell break-in (t = 0 in C) or after store depletion (t = 100 s in D). P < 0.001. **(F and G)** FRET measurements in HEK293 cells transfected with CFP-CAD together with either ORAI-WT-YFP, ORAI1-H134P-YFP, or ORAI1-L273D-YFP. **(F)** Representative confocal images from a total of 52 cells (WT), 22 cells (L273D), and 43 cells (H134P). Scale bars are 5 µm. **(G)** Quantification of FRET efficiencies. P ≤ 0.0001; 0.519. Statistical significance was calculated using ordinary one-way ANOVA (A–D) and one-way ANOVA followed by unpaired Student’s *t* test (E and G). ns, not significant, *P < 0.05, **P < 0.01, ***P < 0.001. AUC, area under the curve; I-V, current–voltage.

**Figure S3.**
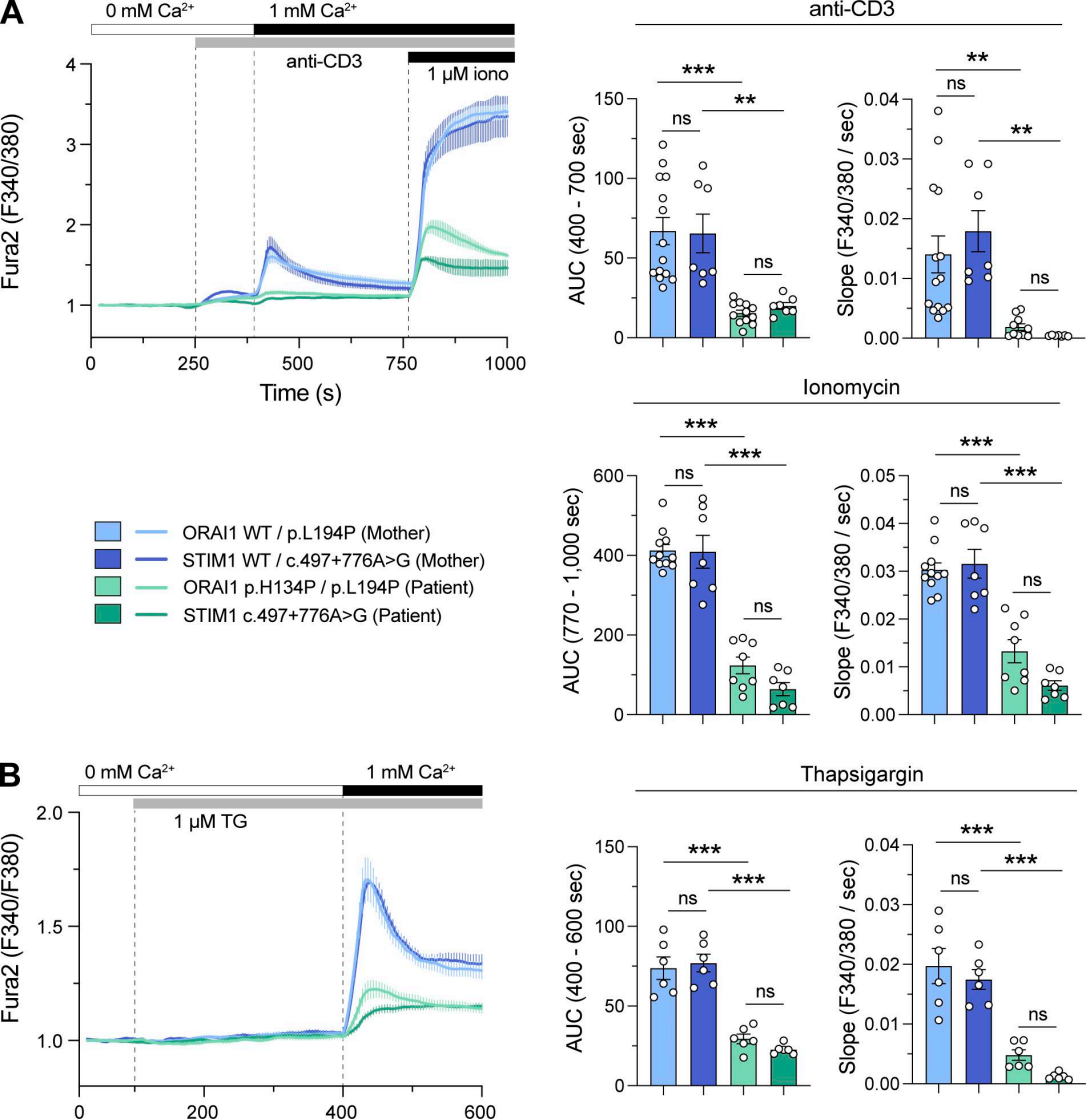
**Strong SOCE defects in T cells of ORAI1 and STIM1 mutant patients. (A and B)** Calcium measurements in *in vitro*–expanded T cells from the ORAI1 mutant patient, his heterozygous mother, a patient with STIM1 LOF mutation (*STIM1 c.497+776A>G*), and his heterozygous mother using Fura-2 AM. Representative time course of Fura-2 fluorescence ratios (340/380 nm) over time (left). Quantification of the AUC and slope of Ca^2+^ influx (right). Data are from at least three independent experiments. **(A)** Cells were placed in 0 mM Ca^2+^ Ringer’s solution, and SOCE was induced by anti-CD3 cross-linking, followed by the addition of 1 mM Ca^2+^ Ringer’s solution, and stimulation with 1 µM ionomycin (iono). For anti-CD3 AUC: P = 0.9991; <0.0001; 0.0049; 0.9774. For anti-CD3 slope: P = 0.7349; 0.0051; 0.0017; 0.9838. For iono AUC: P = 0.9997; <0.0001; <0.0001; 0.3427. For iono slope: P = 0.9685; <0.0001; <0.0001; 0.1042. **(B)** Cells were placed in 0 mM Ca^2+^ Ringer’s solution, and SOCE was induced by stimulation with 1 µM TG followed by the addition of 1 mM Ca^2+^. AUC: P = 0.9633; <0.0001; <0.0001; 0.7556. Slope: P = 0.7963; <0.0001; <0.0001; 0.4852. Statistical significance was calculated using ordinary one-way ANOVA. ns, not significant, **P < 0.01, ***P < 0.001. AUC, area under the curve.

We next measured SOCE in T cells of the patient and his parents. TCR stimulation by anti-CD3 cross-linking resulted in robust SOCE in the presence of extracellular Ca^2+^ in T cells of the father (p.H134P/WT) and the mother (p.L194P/WT), whereas Ca^2+^ entry was significantly suppressed in the patient’s T cells (p.H134P/p.L194P) (Fig. 3 A). Subsequent stimulation with the Ca^2+^ ionophore ionomycin following TCR stimulation to completely deplete ER Ca^2+^ stores and maximally activate SOCE resulted in significantly reduced Ca^2+^ influx in the patient’s T cells compared with strong SOCE in cells of the parents. Moreover, the depletion of ER Ca^2+^ stores with the sarco/ER Ca^2+^ ATPase inhibitor thapsigargin (TG) also revealed a strong defect in SOCE in the patient’s T cells compared with those of his parents (Fig. 3 B). Of note, the reduced SOCE in T cells from the ORAI1 mutant patient in response to either CD3 cross-linking, ionomycin, or TG stimulation was slightly less severe than that observed in T cells of a previously described patient with LOF mutation in *STIM1* that abolishes STIM1 protein expression (Fig. S3, A and B) (20). Together, these data show that compound heterozygosity for both *ORAI1* mutations strongly suppresses stimulation-induced SOCE.

We therefore directly assessed CRAC channel function by whole-cell patch-clamp electrophysiology, focusing on the effects of the p.H134P mutation for several reasons. First, the p.L194P mutation abolished ORAI1 protein expression, making it unlikely to support measurable CRAC currents. Second, the H134 residue, located in TM2, has been shown to play a critical role in stabilizing the closed conformation of the ORAI1 channel. Specifically, H134 functions as a steric brake at the nexus of the TM1–TM2–TM3 interface, and its interaction with nearby residues helps maintain the channel in a closed state (21). Disruption of this intrahelical interaction through small or flexible amino acid substitutions, such as alanine or proline, destabilizes the closed conformation and can result in a partially or fully constitutively open channel (21). Consistent with this model, the H134A mutation (H206A in *Drosophila*) was used to determine the first open-state crystal structure of ORAI1 (22). Third, SOCE was only partially reduced in the patient’s T cells suggesting the presence of residual CRAC channel activity. To study the functional properties of the ORAI1 p.H134P mutant, we expressed it in HEK293 cells either alone or in combination with STIM1. When expressed alone, ORAI1-H134P resulted in a modest but constitutively active inward current with a reversal potential around +48 mV, consistent with a Ca^2+^-selective CRAC current (Fig. 3 C). The current exhibited typical CRAC channel features, including inhibition by lanthanum (La^3+^) and 2-APB, and transient permeation of Na^+^ ions in divalent-free (DVF) conditions. The coexpression of STIM1 did not enhance the baseline constitutive current (Fig. 3 D). Moreover, the constitutive Ca^2+^ current was not further increased when ER Ca^2+^ stores in HEK293 cells were depleted with BAPTA to activate STIM1 and, thus, CRAC channel opening, which was in contrast to cells coexpressing ORAI1-WT and STIM1 (Fig. 3, D and E). Together, these findings indicate that the ORAI1-H134P mutant forms a constitutively active (although not fully open), STIM1-insensitive CRAC channel. The results support a model in which H134 acts as a critical structural element in ORAI1 channel function, and that substitution with proline disrupts its stabilizing role, leading to impaired channel closure and uncoupling of STIM1 gating.

Given that the coexpression of STIM1 failed to enhance the constitutive Ca^2+^ current in cells expressing the ORAI1-H134P mutant, we asked whether this impaired activation might arise from a defect in STIM1 binding. To address this possibility, we examined the interaction between ORAI1 and the CRAC activation domain (CAD) of STIM1 using fluorescence resonance energy transfer (FRET)–based imaging in HEK293 cells (23). CAD is a short 107–amino acid soluble protein that binds to the C terminus of ORAI1 and is sufficient to activate the channel (23, 24, 25). In cells coexpressing ORAI1-WT (YFP) and CFP-CAD, both fusion proteins localized predominantly near or at the PM (Fig. 3 F). Likewise, ORAI1-H134P-YFP and CFP-CAD colocalized near the PM in the majority of cells. Note that in the small subset of cells with intracellular ORAI1-H134P localization, CAD also remained cytoplasmic. The proximity of ORAI1 and CAD was confirmed by the robust FRET signal we observed between ORAI1-WT-YFP and CFP-CAD, as well as ORAI1-H134P-YFP and CFP-CAD (Fig. 3 G), which is consistent with effective binding of CAD to the mutant ORAI1-H134P protein. As a negative control, we examined cells coexpressing CFP-CAD and ORAI1-L273D-YFP. L273 is located in the C-terminal coiled-coil domain of ORAI1 and is critical for STIM1 binding and, thus, gating of ORAI1 channels. Mutation of L273D has been shown to prevent binding of CAD to ORAI1 (26). Accordingly, we find that CAD (CFP) is located in the cytoplasm, and not in or near the PM, when coexpressed with ORAI1-L273D (Fig. 3 F) and that FRET between CAD (CFP) and ORAI1-L273D-YFP is abolished (Fig. 3 G). In contrast, ORAI1-H134P retained the ability to bind CAD at levels comparable to ORAI1-WT (Fig. 3, F and G). These results indicate that the failure of STIM1 to further activate the ORAI1-H134P channel is not due to a loss of STIM1-ORAI1 binding. Instead, they support the conclusion that the H134P mutation disrupts a critical conformational step in the gating mechanism downstream of STIM1 binding. This is consistent with our electrophysiological findings that ORAI1-H134P forms a constitutively active, but not fully open, CRAC channel that is uncoupled from STIM1-mediated gating.

### Altered frequencies of T and NK cell subsets in ORAI1-deficient patient

**Figure 4.**
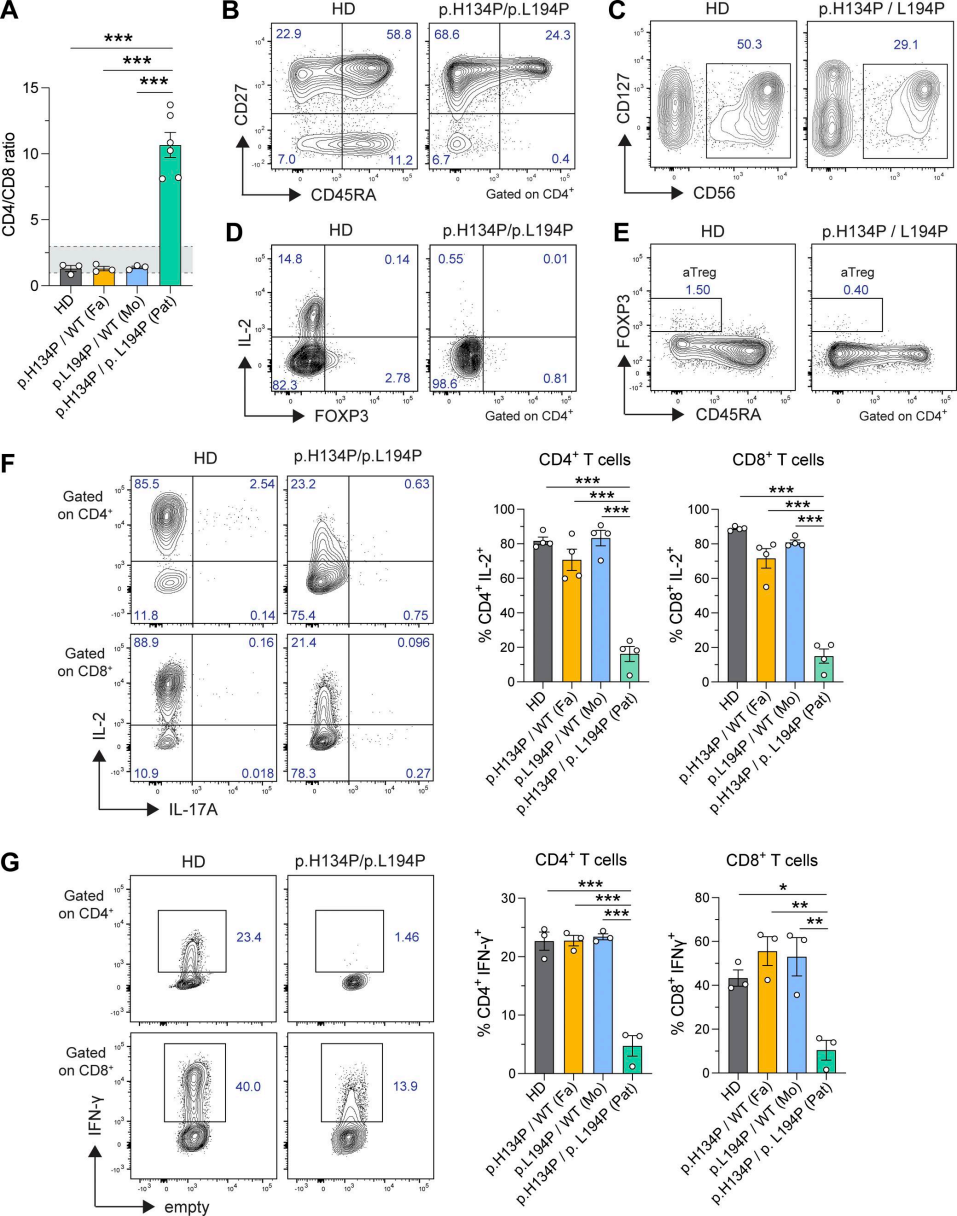
**Altered immune cell frequencies and impaired cytokine production by T cells of ORAI1 mutant patient. (A)** CD4/CD8 T cell ratio in PBMCs of HD, patient (Pat), his father (Fa), and mother (Mo). Data are from six independent experiments. P < 0.0001. **(B)** Flow cytometry analysis of CD27 and CD45RA expression on CD4^+^ T cells within PBMC of an HD control and the patient. **(C)** Flow cytometry analysis of CD127 and CD56 expression on NK cells within PBMC of an HD and the patient. **(D)** Flow cytometry analysis of IL-2 production and FOXP3 expression by CD4^+^ T cells within PBMC of an HD or the patient. Cells were stimulated with PMA/ionomycin for 5 h followed by intracellular antibody staining. **(E)** Flow cytometry analysis of intracellular FOXP3 and surface CD45RA expression in CD4^+^ T cells within PBMC of an HD or patient. **(F and G)**, Cytokine production by in vitro–expanded T cells stimulated with PMA/ionomycin for 4 h. Representative flow cytometry plots (left) and quantification (right). **(F)** IL-2 production by CD4^+^ and CD8^+^ T cells. Data are from four independent experiments. P < 0.0001. **(G)** IFN-γ production by CD4^+^ and CD8^+^ T cells. Data are from three independent experiments. For CD4^+^ T cells: P < 0.0001. For CD8^+^ T cells: P = 0.0232; 0.0038; 0.0055. Statistical significance was calculated using ordinary one-way ANOVA. *P <0.05, **P <0.01, ***P <0.001.

**Figure S4.**
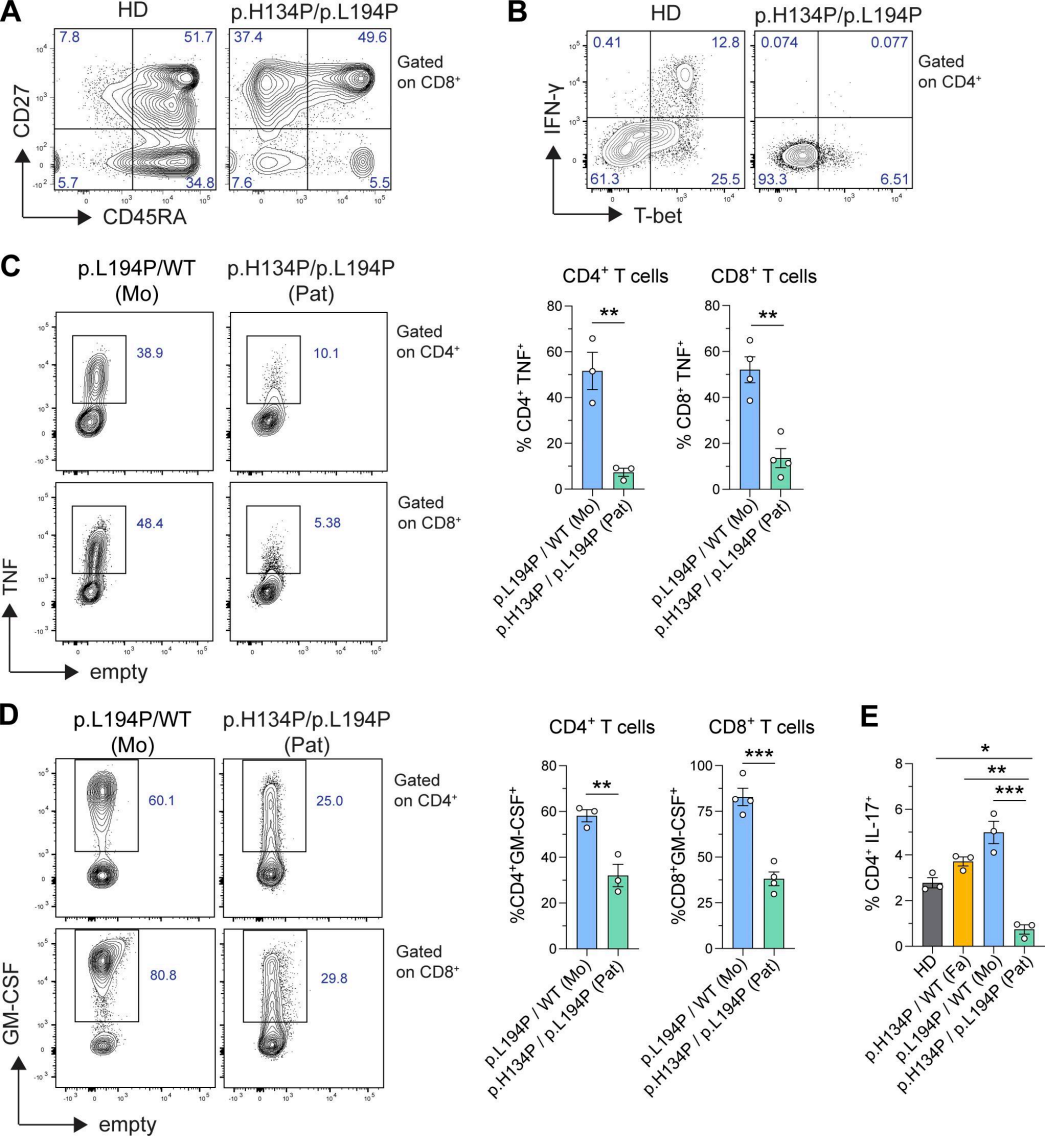
**Reduced cytokine production by CD4** ^**+**^ **and CD8** ^**+**^ **T cells of the patient with ORAI1 mutation. (A)** Flow cytometry analysis of CD27 and CD45RA expression on CD8^+^ T cells within PBMC of an HD control and the patient. **(B)** Flow cytometry analysis of IFN-γ production and T-bet expression of CD4^+^ T cells within PBMC from an HD and the patient. Cells were stimulated with PMA/ionomycin for 5 h. **(C)** TNF production in in vitro–expanded T cells from the patient (Pat) and his mother (Mo) stimulated with PMA/ionomycin for 4 h. Representative flow cytometry plot (left) and quantification (right). For CD4^+^ T cells: P = 0.006. For CD8^+^ T cells: P = 0.0016. **(D)** GM-CSF production in in vitro–expanded T cells from the patient (Pat) and his mother (Mo) stimulated with PMA/ionomycin for 4 h. Representative flow cytometry plot (left) and quantification (right). For CD4^+^ T cells: P = 0.0091. For CD8^+^ T cells: P = 0.0003. **(E)** IL-17 production in in vitro–expanded CD4^+^ T cells from the HD, the patient (Pat), his father (Fa), and his mother (Mo) stimulated with PMA/ionomycin for 4 h (representative flow cytometry plots are shown in Fig. 4 F). P = 0.0212; 0.0011; 0.0001. Data in C–E are from three to four independent experiments for CD4^+^ and CD8^+^ T cells, respectively. Statistical significance was calculated using an unpaired *t* test in C and D and ordinary one-way ANOVA in E. *P < 0.05, **P < 0.01, ***P < 0.001.

Overall lymphocyte levels, including those of CD4^+^ T cells and B cells, were normal in the patient’s blood, although a moderate reduction of CD8^+^ T cells was noted (Table 1). Confirming this finding, the CD4/CD8 T cell ratio was increased in the patient compared with an HD control and both parents (Fig. 4 A). Flow cytometry analysis of peripheral blood mononuclear cells (PBMCs) from the patient and an HD showed markedly increased frequencies of CD4^+^ and CD8^+^ central memory T cells (CD45RA^−^ CD27^+^) and an almost complete absence of CD4^+^ and CD8^+^ terminally differentiated effector memory T (TEMRA, CD45RA^+^ CD27^−^) cells in the patient’s blood (Fig. 4 B and Fig. S4 A). These findings indicate a shift away from highly cytotoxic TEMRA cells to central memory T cells. The patient’s clinical evaluation had shown low normal levels of NK cells in his blood and impaired NK cell function (Table 1). Our flow cytometry analysis confirmed reduced frequencies of CD56^+^ NK cells in his PBMC (Fig. 4 C). The patient also showed decreased proportions of FOXP3^+^ regulatory T (Treg) cells (Fig. 4 D) consistent with reports in other patients with *ORAI1* or *STIM1* LOF mutations (12, 27, 28). A further characterization of Treg cells revealed a decrease in the frequencies of FOXP3^+^ CD45RA^–^-activated Treg cells (Fig. 4 E). Taken together, the reduced frequencies of cytotoxic TEMRA and NK cells are suggestive of reduced acute cytotoxic effector functions against pathogens, and provide a potential explanation for the patient’s HLH phenotype.

### ORAI1 deficiency impairs cytokine production by CD4^+^ and CD8^+^ T cells

CRAC channels and SOCE are essential for T cell function (1, 5). T cells from the patient showed reduced proliferation upon phytohemagglutinin (PHA) stimulation in vitro (Table 1), consistent with previous reports about impaired proliferative responses of ORAI1- and STIM1-deficient T cells (9). To further investigate the functional effects of the patient’s ORAI1 mutations, we measured cytokine production of in vitro–cultured CD4^+^ and CD8^+^ T cells upon stimulation with phorbol 12-myristate 13-acetate (PMA) and ionomycin (P/I). Compared with an HD control and both parents, CD4^+^ and CD8^+^ T cells of the patient showed reduced production of IL-2 and IFN-γ (Fig. 4, F and G). A similar reduction in IFN-γ production was observed in CD4^+^ T cells isolated from the patient’s PBMC compared with those of an HD control after P/I stimulation (Fig. S4 B). The patient’s CD4^+^ T cells also showed reduced expression of the transcription factor T-bet compared with an HD control (Fig. S4 B), indicative of impaired differentiation of CD4^+^ T cells into T helper 1 (Th1) cells, which is consistent with a recent report of a STIM1-deficient patient (20). Besides IFN-γ, we also observed reduced TNF and GM-CSF production by in vitro–expanded CD4^+^ and CD8^+^ T cells from the patient compared with his mother (Fig. S4, C and D). Moreover, IL-17A levels were decreased in the patient’s CD4^+^ T cells compared with an HD and both parents (Fig. 4 F and Fig. S4 E). Taken together, these data demonstrate that T cells from the patient with ORAI1 p.H134P/L194P mutation have a severe defect in P/I-induced cytokine production.

### Enhanced CD4^+^ T cell and NK cell activation accompanied by CD8^+^ T cell dysfunction

**Figure S5.**
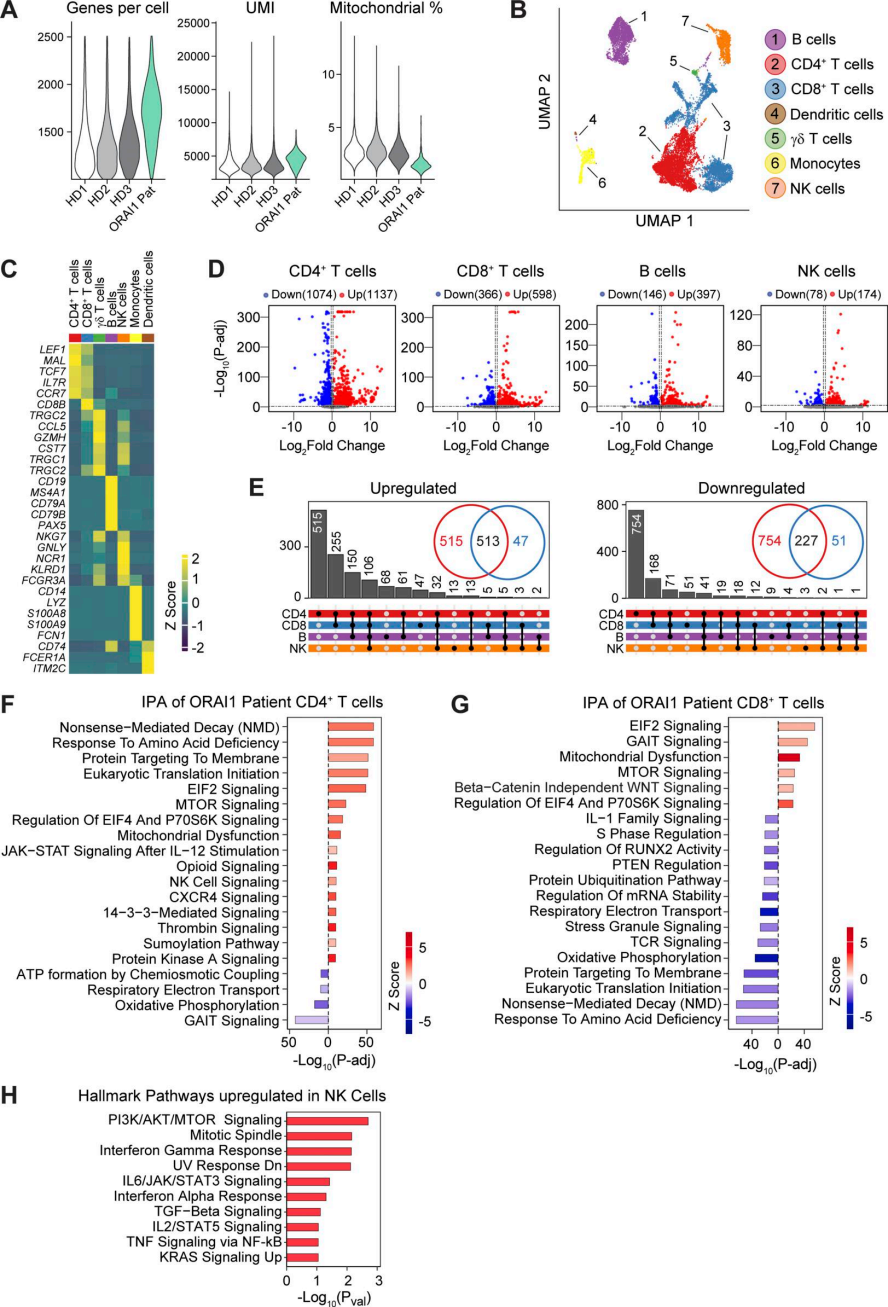
**Enhanced T cell activation in ORAI1 mutant patient.** scRNA-seq of PBMC from the ORAI1 mutant patient and three HD controls combined. **(A)** Violin plots for quality control metrics for each sample after filtering and integration. UMI, unique molecular identifier. **(B)** Aggregate UMAP plot of immune cell subsets identified in 15,000 PBMC from the patient and three controls. **(C)** Heatmap of conserved canonical marker genes of seven distinct major immune cell subsets. **(D)** Volcano plots of DEGs in the patient and three HD controls for each identified cell type. **(E)** Upset plot of the overlap between DEGs in different cell types and a Venn diagram representing the corresponding overlap between CD4^+^ and CD8^+^ T cells. **(F and G)** Top 20 dysregulated pathways based on DEGs in the patient and HD controls using the IPA platform in CD4^+^ T cells (F) and CD8^+^ T cells (G). **(H)** Top 10 upregulated Hallmark pathways in NK cells of the patient. Z scores in C were calculated using the normalized average expression per cell in each cell type across all samples. Statistical analysis in D was performed using a Wilcoxon rank sum test. Genes in E were considered significant if the P_adj_ was <0.05. The significance of pathways shown in F–H was calculated using right-tailed Fisher’s exact test. The significance in D, F, and G was adjusted using the Benjamini–Hochberg method.

To further understand the immune dysfunction in the ORAI1-deficient patient, we conducted single-cell RNA sequencing (scRNA-seq) of unstimulated PBMC from the patient and three HD controls. After quality control filtering and batch correction, we identified seven major mononuclear cell populations including CD4^+^ and CD8^+^ T cells, B cells, γδ T cells, NK cells, dendritic cells, and monocytes (Fig. S5, A–C). To identify the effects of ORAI1 deficiency on the functional state of immune cells, we compared differentially expressed genes (DEGs) in the patient and HD controls for each major cell type (with the exception of monocytes, whose abundance was too low in the patient’s PBMC sample for analysis). Whereas the numbers of DEGs were relatively small in B and NK cells, we observed >2,200 and >950 DEGs in CD4^+^ and CD8^+^ T cells, respectively (Fig. S5 D). The majority of DEGs in CD8^+^ T cells were also differentially expressed in CD4^+^ T cells, whereas the overlap between DEG in T cells and other cell types was minimal (Fig. S5 E). A pathway analysis of DEGs showed that a subset of pathways were dysregulated in a similar manner in both CD4^+^ and CD8^+^ T cells of the patient and either enriched (including mTOR signaling) or depleted (for instance, oxidative phosphorylation and respiratory electron transport, Fig. S5, F and G). Other pathways were altered in opposite directions in the patient’s CD4^+^ and CD8^+^ T cells. For instance, his CD4^+^ T cells showed an enrichment of pathways related to protein translation, whereas IFN-γ–activated inhibitor of translation (GAIT) signaling was depleted (Fig. S5 F). The same pathways were inversely regulated in the patient’s CD8^+^ T cells (Fig. S5 G). As in CD4^+^ and CD8^+^ T cells, we also observed a significant upregulation of many DEGs in NK cells of the patient. A pathway analysis revealed increased signaling through several cytokine receptors including those for IL-2, IL-6, TNF, and TGF-β, as well as enhanced IFN-α and IFN-γ responses (Fig. S5 H), indicative of a substantial activation of NK cells in the patient despite his impaired SOCE.

**Figure 5.**
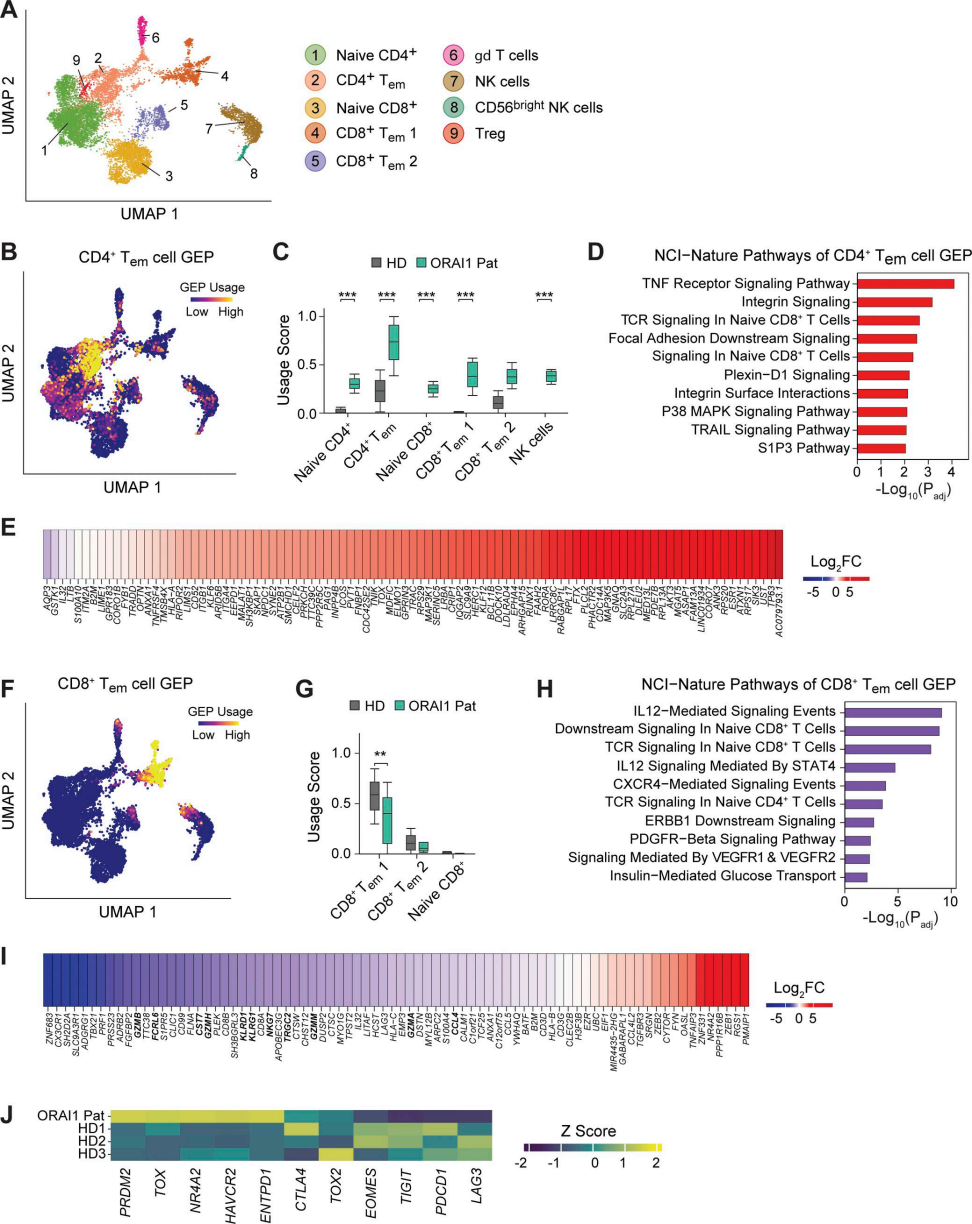
**Enhanced CD4** ^**+**^ **T cell activation and CD8** ^**+**^ **T cell dysfunction in the ORAI1 mutant patient.** scRNA-seq analysis of PBMC from the *ORAI1* mutant patient and three HD controls combined. **(A)** Aggregate UMAP plot of T-NK cell subsets identified in PBMC of the patient and HD controls. **(B)** UMAP plot of PBMC colored by the expression of the GEP associated with *CD4*^*+*^*T*_*em*_*cells* identified by NMF. **(C)** Boxplot showing usage scores for this GEP in CD4^+^ and CD8^+^ T cells, and NK cells. P < 0.001. **(D)** Top 10 dysregulated National Cancer Institute (NCI)-Nature pathways in the *CD4*^*+*^*T*_*em*_*cell* GEP. **(E)** Heatmap of DEGs contained in the *CD4*^+^*T*_*em*_*cell* GEP in all CD4^+^ T cells of the patient compared with HD controls. Colors depict log_2_FC in expression. **(F)** UMAP plot of PBMC colored by the expression of the GEP associated with *CD8*^*+*^*T*_*em*_*cells* identified by NMF. **(G)** Boxplot showing usage scores for this GEP in naive and T_em_ CD8^+^ T cells. P < 0.01. **(H)** Top 10 dysregulated NCI-Nature pathways in the *CD8*^*+*^*T*_*em*_*cell* GEP. **(I)** Heatmap of DEGs contained in the *CD8*^*+*^*T*_*em*_*cell* GEP in all CD8^+^ T cells of the patient compared with HD controls. Colors depict log_2_FC in expression. **(J)** Heatmap of exhaustion markers in CD8^+^ T cells from three HD controls and the patient. Statistical analyses in D and H were conducted using a right-tailed Fisher’s exact test. Statistical analyses in C and G were performed using a Wilcoxon rank sum test. Z scores in J were calculated using the normalized average expression per sample in all CD8^+^ T cells. Significance in C, D, G, and H was adjusted using the Benjamini–Hochberg method. **P < 0.01, ***P < 0.001. FC, fold change.

The analysis of DEGs suggested an activated state of CD4^+^ T cells in the ORAI1-deficient patient, whereas his CD8^+^ T cells showed signs of both activation and suppression. Enhanced CD4^+^ T cell activation was unexpected because SOCE mediated by ORAI1 is required for T cell activation and functions such as proliferation and cytokine production (1, 4, 5). Because the DEG and pathway analyses did not provide detailed insights into which subsets of CD4^+^ and CD8^+^ T cells were dysregulated in the ORAI1-deficient patient, we used a machine learning approach to refine our analysis of the scRNA-seq data. We employed nonnegative matrix factorization (NMF) to identify gene expression profiles (GEPs) correlating with distinct T cell subsets, which might provide additional insights into the functional states of the patient’s lymphocytes. Using cell-type identification by marker genes, we detected nine subtypes of T and NK cells including naive and memory CD4^+^ and CD8^+^ T cells, as well as γδ T cells and Treg cells (Fig. 5 A).

By conducting an NMF analysis of all T and NK cell subsets, we identified nine GEPs with mean usage scores of >0.1 for at least one cell type. Two of these GEPs overlapped with distinct T cell subpopulations and were differentially used by the patient’s T cells compared with HD controls (Fig. 5, B–J). In contrast, no GEPs associated with Treg cells, γδ T cells, or NK cells were detected. The first of the two GEPs was utilized predominantly by CD4^+^ T effector memory (T_em_) cells and hence called *CD4*^*+*^*T*_*em*_*cell* GEP (Fig. 5 B). Its usage was most strongly increased in CD4^+^ T_em_ cells of the ORAI1-deficient patient, but was also enhanced in other patient T cell subsets and NK cells (Fig. 5 C). A pathway analysis of genes contained in this GEP identified a significant enrichment of TNF receptor, integrin, TCR, and other signaling pathways in the patient (Fig. 5, D and E). Signs of increased immune signaling are present despite impaired SOCE and may be secondary to the patient’s persistent viral infection as it not only affected CD4^+^ T_em_ cells, but also CD8^+^ T cells and NK cells. The second GEP we identified was predominantly utilized by one of two subsets of CD8^+^ T effector cells (CD8^+^ T_em_ 1), but not naive or CD8^+^ T_em_ 2 cells (Fig. 5 F). Its usage was significantly reduced in the patient’s CD8^+^ T_em_ 1 cells (Fig. 5 G). A pathway analysis of the genes included in this *CD8*^*+*^*T*_*em*_*cell* GEP showed the depletion of IL-12, TCR, and other signaling pathways (Fig. 5 H). A closer analysis of the *CD8*^*+*^*T*_*em*_*cell* GEP revealed a strong downregulation of genes related to the cytotoxic function of CD8^+^ T cells (Fig. 5 I). These included *GZMA*, *GZMB*, *GZMH*, and *GZMM* (encoding the serine protease granzymes A, B, H, and M required for CD8^+^ T cell–mediated cytotoxicity [29]) and *NKG7* (encoding natural killer cell granule protein 7, which enhances CD8^+^ T cell immune synapse efficiency and promotes cytotoxicity [30]). These findings suggest that the patient’s CD8^+^ T cells may have reduced cytotoxic function. Given the depletion of the *CD8*^*+*^*T*_*em*_*cell* GEP in the patient’s CD8^+^ T cells and his chronic CMV infection, we investigated the expression of genes associated with CD8^+^ T cell exhaustion (31). While the expression of *TOX*, *HAVCR2* (encoding TIM3), and *ENTPD1* (encoding CD39) was upregulated in the patient CD8^+^ T cells, that of other exhaustion markers including *LAG3*, *PDCD1*, and *TIGIT* was reduced (Fig. 5 J). Thus, although the patient’s CD8^+^ T_em_ cells show signs of being dysfunctional, this defect appears to be distinct from T cell exhaustion.

## Discussion

Here, we demonstrate that a patient with compound heterozygous missense mutations in *ORAI1* (p.H134P and p.L194P) has strongly attenuated but not abolished SOCE. One of the mutations, L194P, has been reported previously (12, 13, 14). Whereas one patient was compound-heterozygous for mutations in L194P and A103E (13, 14), another was homozygous for the L194P mutation (12). T cells from the latter patient showed abolished ORAI1 expression at or near the PM and SOCE (12). Here, we show that the ectopic expression of the mutant ORAI1-L194P protein in HEK cells results in its retention in the cytosol with no apparent translocation to the PM region. These findings suggest that mutant ORAI1-L194P protein, although expressed, cannot contribute to CRAC channel function. Accordingly, the residual SOCE in the patient’s T cells likely results exclusively from a channel formed by mutant ORAI1-H134P proteins as the majority of ectopically expressed ORAI1-H134P is localized at the PM. Electrophysiological recordings of ORAI1-H134P–expressing cells revealed two key properties of the mutant channel. First, we observed a constitutive Ca^2+^ current in nonstimulated cells, which had hallmarks characteristic of CRAC channels, indicating that the H134P mutation produces a constitutively open channel albeit with reduced conductance compared with activated WT channels. Second, the constitutive current did not increase as expected when the ORAI1-H134P mutant was coexpressed with STIM1 (compared with the expression of ORAI1-H134P alone) despite intact STIM1 binding. These findings indicate that the mutant ORAI1-H134P channel exhibits both GOF and LOF properties that manifest as constitutive activity in the absence of stimulation but impaired STIM1-dependent gating, respectively.

A mutation at position H134 of ORAI1 in human patients has not been reported before. Engineered mutations of H134, however, have demonstrated that H134 is critically important for CRAC channel function, particularly channel gating (21). H134A/C/S/T mutant channels display strong constitutive inward-rectifying Ca^2+^-selective currents in the absence of STIM1 that do not increase when STIM1 is coexpressed (21, 32). In contrast, H134V/E/M/Q channels are only partially active without STIM1, and STIM1 coexpression boosts current amplitudes. These and additional data indicate that the constitutive activation of certain ORAI1-H134 mutants results from a decrease in the side-chain volume, which releases a “brake” at the TM1–TM2 domain interface and enables the channel to open. Conversely, increasing the volume of the side chain inhibits STIM1’s ability to gate the channel. It is important to note that the TM1 pore helix forms distinct functional contacts with surrounding helices including TM2 (in which H134 is located) and TM3. We speculate that the patient’s H134P mutation perturbs the alpha helical structure of TM2 (as proline mutations are known to disrupt alpha helices [33]) and thereby weakens TM1 and TM2 interactions, locking the ORAI1 channel in a semi-open conformation, which is unresponsive to channel opening by STIM1-mediated gating. Thus, the H134P mutation is not simply disrupting the alphahelical structure of TM2 but interferes more specifically with a critical gating mechanism of the ORAI1 channel.

The patient’s clinical presentation as CRAC channelopathy with immunodeficiency is likely explained by several aspects of his H134P and L194P mutations in ORAI1. First, the L194P mutant protein exhibits defective trafficking to the PM, resulting in functional CRAC channels being composed solely of ORAI1-H134P subunits. However, because ORAI1-H134P also has a mild PM trafficking defect, the actual number of functional ORAI1 channel numbers in the PM is likely far below 50% compared with healthy controls. Moreover, the ORAI1-H134P channel, although partially constitutively open, cannot be activated by STIM1 binding, resulting in impaired Ca^2+^ influx following TCR activation. As a result, the patient’s cells fail to effectively use SOCE to promote the expression of cytokines and engage other Ca^2+^-dependent cellular immune responses. The physiological implication of these findings is that partial constitutive opening of CRAC channels without further activation by STIM1 is not sufficient for productive T cell–mediated immune responses. Of note, GOF mutations in ORAI1 resulting in constitutively open CRAC channels (including p.G98S, p.L138F, p.V107M, and p.T184M) have been reported and cause TAM or Stormorken syndrome (9, 10). These patients, however, are not immunodeficient, likely because their ORAI1 channels retain the ability to be gated by STIM1 and, thus, to mediate Ca^2+^ influx in response to immunoreceptor stimulation (34, 35).

The patient’s immunodeficiency and his other non–immune-related symptoms including thermoregulatory instability (indicative of anhidrosis) and muscular hypotonia are consistent with CRAC channelopathy disease reported in other patients with LOF mutations in *ORAI1* and *STIM1* genes (9, 36). Functionally, the patient’s CD4^+^ and CD8^+^ T cells were characterized by a strongly reduced production of a variety of cytokines including IL-2, IFN-γ, and TNF (as well as GM-CSF and IL-17 in CD4^+^ T cells). These defects are reminiscent of the attenuated T cell function reported in other *ORAI1*- and *STIM1*-deficient patients (9, 12, 36) and likely contribute to the patient’s severe immunodeficiency. His CD4^+^ and CD8^+^ T cells were also characterized by an almost complete loss of TEMRA cells and increased proportions of central memory T cells. These findings suggest that the patient’s T cells have undergone antigen-specific activation and differentiation into long-lived memory cells, potentially in response to his chronic CMV infection. They also indicate that some aspects of T cell function are intact despite severely reduced SOCE and functional impairments such as decreased cytokine production and proliferation. The preserved, and in fact increased, differentiation of the patients’ CD4^+^ and CD8^+^ T cells into memory cells for instance is likely driven by pathways such as cytokine and interferon receptor signaling that are independent of SOCE.

To investigate the immune dysregulation in the ORAI1-deficient patient, we conducted scRNA-seq of his PBMC. Gene expression was both up- and downregulated in his immune cells with the largest numbers of DEGs observed in CD4^+^ and CD8^+^ T cells. The analysis of DEGs showed that many pathways were upregulated in CD4^+^ T cells including those associated with protein translation, whereas signaling through the GAIT complex was reduced. This is intriguing because the GAIT complex targets 3′ untranslated regions of mRNAs encoding inflammatory proteins such as *CCL2*, *S100A8*, and *TAP1* in macrophages and suppresses their translation (37). Decreased GAIT signaling in CD4^+^ T cells of the ORAI1-deficient patient may be indicative of their increased proinflammatory function. Further investigation of the mechanisms underlying the dysregulated gene expression in CD4^+^ T cells of the ORAI1-deficient patient using machine learning–based NMF identified a GEP associated with CD4^+^ T_em_ cells whose usage was significantly increased in the patient. Genes contained in this *CD4*^*+*^*T*_*em*_*cell* GEP were linked to TNF receptor, integrin, TCR, and other signaling pathways. These findings indicate that the patient’s CD4^+^ T_em_ cells are activated despite severely impaired SOCE, which we speculate to be secondary to his chronic CMV infection and mediated by Ca^2+^-independent signaling pathways. The increased T cell activation observed by transcriptomic analysis contrasts with the reduced production of Ca^2+^-dependent cytokines we had detected by flow cytometry. It is important to note that PBMC had not been stimulated prior to scRNA-seq in contrast to the cytokine analyses, thus resulting in a potential underestimation of the SOCE defect on gene expression in the transcriptomic data.

In contrast to increased activation of the patient’s CD4^+^ T_em_ cells, his CD8^+^ cells appear to be dysfunctional. This assessment is based on the reduced cytokine production after CD8^+^ T cell stimulation, and the downregulation of gene expression and associated pathways in CD8^+^ T cells. The latter included protein translation, metabolism, and TCR signaling. Moreover, NMF identified a unique GEP that was predominantly used by a subset of CD8^+^ T_em_ cells and significantly underutilized in the patient. This *CD8*^*+*^*T*_*em*_*cell* GEP contains many genes related to the cytotoxic function of CD8^+^ T cells including several that encode granzymes and NKG7. The depletion of these genes and the *CD8*^*+*^*T*_*em*_*cell* GEP suggests that the patient’s CD8^+^ T cells may have reduced cytotoxic function. The causes underlying this potential dysfunction could be related to reduced SOCE and, in addition, an altered cellular state resembling CD8^+^ T cell exhaustion secondary to chronic viral infection (31). A study of a STIM1-deficient patient had indeed found elevated levels of inhibitory receptors associated with T cell exhaustion (27). Arguing against CD8^+^ T cell exhaustion in our patient, however, is the variegated expression pattern of exhaustion markers in his CD8^+^ T cells.

The ORAI1-deficient patient presented with symptoms of HLH that were characterized by hepatosplenomegaly, anemia, thrombocytopenia, hyperferritinemia, and hypertriglyceridemia. A key factor underlying the pathogenesis of HLH and the associated hyperinflammatory phenotype is the impaired cytotoxicity of CD8^+^ T cells and NK cells (38). Defective killing of virus-infected cells by CD8^+^ T cells maintains the immune response to infection and results in increased IFN-γ production, which is further exacerbated by the reduced cytotoxic function of NK cells and their ability to eliminate activated CD8^+^ T cells. The importance of IFN-γ for HLH pathology is emphasized by the successful use of emapalumab, a monoclonal antibody targeting IFN-γ, for the treatment of refractory HLH (39). The impaired NK cell cytotoxicity (Table 1) and increased IFN-γ signaling (Fig. S5 H) of our patient may have contributed to his HLH in the context of chronic CMV infection. Indeed, impaired NK cell function and HLH have been observed in another patient with LOF mutation in *ORAI1* (40). Moreover, the reduced frequencies of cytotoxic CD8^+^ TEMRA cells in the patient, and the depletion of cytotoxicity-associated genes including granzymes in the *CD8*^*+*^*T*_*em*_*cell* GEP have likely contributed to his HLH phenotype. Although IFN-γ production in response to TCR stimulation is impaired in the ORAI1-deficient patient, other signaling pathways promoting IFN-γ production are not dependent on SOCE. These include signaling through IL-12 and IL-18 receptors and Toll-like receptors. In fact, we recently showed that suppression of SOCE in mouse and human CD4^+^ T cells increases the expression of IL-12 receptor β1 and β2, thus sensitizing the cells to IL-12 signaling and promoting SOCE-independent IFN-γ expression (20).

Here, we show that a unique H134P mutation in ORAI1 is associated with immunodeficiency and HLH. The complex cellular immunological phenotype in the patient mirrors the mixed LOF and GOF properties of the mutant ORAI1 channel, which is constitutively, but not fully, open and at the same time unresponsive to activation by STIM1. The physiological implication of our findings is that a partially constitutively active CRAC channel that is unresponsive to antigen receptor–induced opening is not sufficient for T cell activation and immunity to infection.

## Materials and methods

### Patient

A 4-mo-old male Caucasian infant from a nonconsanguineous family presented with pneumococcal sepsis requiring resuscitation and intensive care admission. Treatment with intravenous antibiotics resulted in clinical improvement; however, he had persistent fevers and developed hepatosplenomegaly, anemia (hemoglobin 74 g/Liter), and thrombocytopenia (platelet count 73 × 10^9^/Liter) with mildly elevated ferritin (1,293 µg/Liter) consistent with HLH. Further investigations revealed a very high CMV load in the peripheral blood measured by PCR (>1 million copies/Liter), which was associated with CMV retinitis (Fig. S1) and pneumonitis. He was treated with intravenous ganciclovir and methylprednisolone for CMV-induced HLH. His cytopenia and ferritin improved with steroid treatment; however, he developed ganciclovir resistance and was therefore changed to foscarnet. The antiviral treatment improved his retinitis and pneumonitis, but CMV viremia persisted.

Routine immunological investigations revealed normal numbers of CD4^+^ T cells and B cells, low normal or moderately reduced numbers of CD8^+^ T cells, NK cells, and monocytes, and hypergammaglobulinemia (IgG 19 g/Liter, IgA 3.7 g/Liter, IgM 2.06 g/Liter). Because of his markedly reduced T cell proliferation and cytotoxic NK cell function (Table 1) but largely normal lymphocyte numbers, the patient was diagnosed with CID and at 10 mo of age underwent hematopoietic stem cell (HSC) transplantation with a 10/10 CMV-experienced, matched unrelated donor. HSC grafts consisted of sorted CD34^+^ stem cells with the subsequent addition of donor CMV-specific cytotoxic T cells in an attempt to control ongoing CMV infection. The patient received a total of three stem cell infusions due to engraftment failure. The last two infusions were preceded by conditioning with treosulfan and fludarabine. Upon eventual engraftment, the patient succumbed to severe graft-versus-host disease that was associated with massive pulmonary infiltrates with respiratory failure and severe neurological inflammation (CSF neopterin 1,529 nmol/Liter; reference range 6–30 nmol/Liter), likely related to his concurrent and persistent CMV infection.

Besides CID, the patient had persistent generalized muscular hypotonia with a delay in gross motor development. His head lag persisted until 10 mo of age and at 12 mo of age; he was unable to sit unsupported. No muscle biopsy was performed as his hypotonia was initially thought to be related to his severe chronic illness. The patient had episodes of thermoregulatory instability associated with tachycardia and hypertension. Although anhidrosis was not formally tested, sweating was not noted throughout his entire admission. A skin biopsy conducted after HSC transplantation showed a thin epidermis but normal presence and architecture of sweat glands. He was noted to have fine sparse hair. Dentition had not occurred at the time of the patient’s death.

Whole-exome sequencing of the patient’s PBMC revealed two independent mutations in the *ORAI1* gene (p.L194P, p.H134P) for which he is compound-heterozygous, whereas his parents are each heterozygous for one of the mutations. Because of the CID phenotype, his associated nonimmunological symptoms, and the ORAI1 mutations, the patient was diagnosed with CRAC channelopathy. His clinical and immunological phenotype are summarized in Table 1.

### Patient samples

Blood samples were collected from the patient pre-hematopoietic stem cell transplantation, as well as each of the parents with parental consent. At the time of blood collection, the patient had a CMV viral load of >10,000 copies/Liter and was receiving ganciclovir (30 mg bd), but was not on any immunosuppressants. PBMCs were isolated via Ficoll-Paque PLUS (17-1440-02; Cytiva) gradient centrifugation. The collection and study of patient sample were approved by the Sydney Children’s Hospital Network ethics committee (12/SCHN/33).

### Cell culture

Human T cells were expanded from PBMC by culture in RPMI 1640 medium (10-040-cv; Corning) supplemented with 2% human serum (H4522; Sigma-Aldrich), 10 % fetal bovine serum (FBS; F4135; Sigma-Aldrich), 2 mM L-glutamine (25030081; Gibco), 50 U/ml penicillin/streptomycin (15-140-122; Gibco), and 25 mM HEPES (15630080; Gibco) upon stimulation with 1 μg/ml PHA-P (L1668; Sigma-Aldrich) and 50 U/ml recombinant human IL-2 (200-02; PeproTech) in the presence of irradiated buffy coat cells (PBMC: feeder cell ratio of 1:1) and EBV-transformed B cells (PBMC: B cell ratio of 1:10). Cells were maintained at 37°C and 5% CO_2_, at a concentration of 1–2 × 10^6^/ml and supplemented with fresh media containing IL-2. Every 2 wk, buffy coat cells, B cells, PHA-P, and IL-2 were added for propagation of the expanded human T cell lines.

### Genomic DNA sequencing analysis

Genomic DNA was extracted from cells with FlexiGene DNA Kit (51206; Qiagen) followed by a PCR with the following primers flanking ORAI1 exons 1 and 2: exon 1, forward 5′-ACA​ACA​ACG​CCC​ACT​TCT​TGG​TGG-3′ and reverse 5′-TGC​TCA​CGT​CCA​GCA​CCT​C-3′; exon 2, forward 5′-TCT​TGC​TTT​CTG​TAG​GGC​TTT​CTG-3′ and reverse 5′-TCT​CAA​AGG​AGC​TGG​AAG​TGC-3′. PCR products were separated using a 1.5% agarose gel, excised with Gel Extraction Kit (28704; Qiagen), and sequenced directly (Genewiz) by using the following primers: exon 1, forward 5′-AGC​ATG​CAA​AAC​AGC​CCA​GG-3′ and reverse 5′-ACG​GTT​TCT​CCC​AGC​TCT​TC-3′; exon 2, forward 5′-TGA​CAG​GAG​GAG​AGC​TAG​G-3′ and reverse 5′-AAG​AGA​TCC​TCC​TGC​CTT​GG-3′.

### Flow cytometry analysis of T cells and PBMCs

Cells were incubated with anti-mouse CD16/32 blocking antibodies (BE0307; BioXCell) for 10 min. Surface staining was performed using molecules with fluorescently labeled antibodies at room temperature for 15 min in the dark. For intracellular staining, cells were fixed with IC Fixation Buffer (00-8222-49; eBioscience) for 30 min, incubated with permeabilization buffer (00-8333-56; eBioscience), and stained intracellularly for 1 h. For cytokine measurements, cells were stimulated with 20 nM PMA (524400; Calbiochem) and 1 μM ionomycin (407952; Sigma-Aldrich) in the presence of 3 µg/ml brefeldin A (00-4506-51; Invitrogen) for 4–6 h followed by washing of the cells in PBS containing 2% FBS prior to staining. The following antibodies were used: eFluor 450 anti-human CD4 (clone OKT-4, 48-0048-42; eBioscience), PE-Cy7 anti-human CD8 (clone SK1, 344750; BioLegend), APC anti-human IL-2 antibody (clone MQ1-17H12, 17-7029-82; eBioscience), Alexa Fluor 488 anti-human TNF-α antibody (clone Mab11, 53-7349-42; eBioscience), PE anti-human IFN-γ antibody (4S.B3, 502510; BioLegend), APC anti-human GM-CSF antibody (clone BVD2-21C11, 502310; BioLegend), and PE anti-human IL-17A antibody (clone eBio64CAP17, 12-7178-42; eBioscience). Samples were acquired on a LSRII flow cytometer (BD Biosciences) and analyzed using FlowJo software (Tree Star, v 10.10.0).

For the analysis of PBMCs, frozen samples from the patient and controls were thawed and assessed for cell viability with trypan blue and were at least 85% viable. 1 × 10^6^ cells per ml were cultured in RPMI 1640 medium (supplemented with 10% FCS, 2 mM L-glutamine, 100 U/ml penicillin, and 100 μg/ml streptomycin; Thermo Fisher Scientific) at 37°C and 5% CO_2_. Cells were stimulated with 0.1 μM PMA and 1 μM ionomycin (Merck) for 5 h. The protein transport inhibitor monensin (0.67 μl/ml; BD Biosciences) was added 4 h prior to harvesting. Cells were surface-stained with antibodies against CD3 (conjugated with BUV395, cat. no. 564001; BD Biosciences) and CD8 (BV510, cat. no. 563919; BD Biosciences) for 20 min at 4°C. Cells were fixed and permeabilized using Transcription Factor Buffer Set (cat. no. 562574; BD Biosciences), according to the manufacturer’s instructions. Cells were subsequently stained intracellularly with antibodies against FoxP3 (conjugated with FITC, cat. no. 320212; BioLegend), IL-2 (BV711, cat. no. 563946; BD Biosciences), IL-17A (APC, cat. no. 512334), T-bet (PE, cat. no. 561265; BD Biosciences), and IFN-γ (BV711, cat. no. 564039; BD Biosciences) for 30 min at room temperature and washed, before acquisition using BD LSRII at The Westmead Institute for Medical Research.

For the expression analysis of ORAI1 proteins at the cell surface, expanded human T cells from the patient, his parents, and an HD were stained with anti-CD4 and anti-CD8 antibodies, as well as an Alexa Fluor 647–conjugated anti-human ORAI1 mAb (2C1.1) recognizing an epitope in the second extracellular loop of ORAI1 (kind gift of H. McBride; Amgen, Thousand Oaks, California, USA) (17, 18). ORAI1-deficient Jurkat cells described previously (41) served as a specificity control.

### Transfection and flow cytometry analysis of HEK293 cells

HEK293-H cells were grown as previously described (21) and were plated onto poly-L-lysine–coated coverslips 1 day before transfection. All mutant ORAI1 constructs were generated by QuikChange Mutagenesis Kit (Agilent Technologies) in the pEYFP-N1 or pECFP-N1 (Clontech) vectors and confirmed by DNA sequencing. For electrophysiology, ORAI1 constructs were transfected into HEK293 cells either alone (200 ng DNA per coverslip) or together with mCherry STIM1 (100 ng ORAI1 and 500 ng STIM1 DNA per coverslip). For FRET and confocal microscopy experiments, cells were transfected with ORAI1-YFP or with CFP-CAD constructs (200 ng of each DNA per coverslip). For flow cytometry, HEK293 cells were transfected with the indicated ORAI1 constructs (100–300 ng). 48 h later, nonpermeabilized cells were stained with a mouse-anti-human ORAI1 (29A2) mAb that recognizes amino acids 196–234 in the second extracellular loop of hORAI1 (19) followed by wash steps and incubation with a secondary Alexa Fluor 647–conjugated goat-anti-mouse Ab. If not stated otherwise, transfections were performed using Lipofectamine 2000 (Thermo Fisher Scientific) 24–48 h prior to electrophysiology or imaging experiments.

### Ca^2+^ measurements

Expanded human T cells were labeled with 2 µM Fura-2 AM (F1221; Invitrogen) for 30 min in complete RPMI. Cells were attached for 10 min to 96-well imaging plates (353219; Falcon) coated with 0.01% poly-L-lysine (P8920; Sigma-Aldrich) diluted in water. Intracellular Ca^2+^ measurements were performed using a Flexstation 3 fluorescence plate reader (Molecular Devices). Cells were stimulated with 10 μg/ml OKT-3 (clone OKT3, 14-0037-83; eBioscience) followed by 1 µM ionomycin (407952; Sigma-Aldrich). Alternatively, cells were stimulated with 1 μM TG (586005; Calbiochem) in Ca^2+^-free Ringer’s solution (155 mM NaCl, 4.5 mM KCl, 1 mM MgCl_2_, 10 mM D-glucose, and 5 mM Na HEPES) to induce store depletion followed by the addition of 1 mM Ca^2+^ Ringer’s solution to induce SOCE. Fura-2 fluorescence emission ratios (F340/380) were collected at 510 nm following excitation at 340 and 380 nm every 5 s. Ca^2+^ signals were quantified by analyzing the slopes and the integrated Ca^2+^ signal (area under the curve) of F340/380 ratios using GraphPad Prism 10 software. Slopes were calculated using the following formula: $\text{slope}=\left(y_{2}-y_{1}\right)/\left(x_{2}-x_{1}\right)$, where y_1_ is the baseline Fura-2 ratio, y_2_ is the peak Fura-2 ratio, and x_1_ and x_2_ are the corresponding time points (in sec).

### Solutions and chemicals

Standard extracellular Ringer’s solution used for electrophysiological experiments contained 130 mM NaCl, 4.5 mM KCl, 20 mM CaCl_2_, 10 mM tetraethylammonium chloride (TEA-Cl), 10 mM D-glucose, and 5 mM HEPES (pH 7.4 with NaOH). The DVF Ringer’s solution contained 150 mM NaCl, 10 mM HEDTA, 1 mM EDTA, 10 mM TEA-Cl, and 5 mM HEPES (pH 7.4). The internal solution contained 135 mM Cs aspartate, 8 mM MgCl_2_, 8 mM Cs-BAPTA, and 10 mM HEPES (pH 7.2 with CsOH). For FRET and confocal imaging studies, Ringer’s solution contained 2 mM CaCl_2_ and 150 mM NaCl with the other components as above.

### Electrophysiology

Currents were recorded in the standard whole-cell configuration at room temperature on an Axopatch 200B amplifier (Molecular Devices) interfaced to an ITC-18 input/output board (InstruTech) as previously described (21). I_CRAC_ was typically activated by passive depletion of ER Ca^2+^ stores by intracellular dialysis of 8 mM BAPTA. All data were corrected for leak currents collected in 150 µM LaCl_3_. Analysis of current amplitudes was performed by measuring the peak currents during the −100 mV pulse.

### FRET microscopy

HEK293H cells transfected with ORAI1-YFP and CFP-CAD DNA constructs were imaged using wide-field epifluorescence microscopy on an IX71 inverted microscope (Olympus). Cells were imaged with a 60× oil immersion objective (UPlanApo NA 1.40), a 175 W Xenon arc lamp (Sutter), and excitation and emission filter wheels (Sutter) as previously described (21). The microscope-specific bleed-through constants (a = 0.12; b = 0.008; c = 0.002; and d = 0.33) were determined from cells expressing cytosolic CFP or YFP alone. The apparent FRET efficiency was calculated from background-subtracted images using the formalism:

$$E_{FRET}=\frac{F_{c}}{F_{c}+GI_{DD}}$$

where $F_{c}=I_{DA}-aI_{AA}-dI_{DD}$ and *I*_*DD*_, *I*_*AA*_, and *I*_*DA*_ refer to the background-subtracted CFP, YFP, and FRET images, respectively. The instrument-dependent *G* factor had the value 1.85 ± 0.1. E-FRET analysis was restricted to cells with YFP/CFP ratios in the range of 2–6 to ensure that E-FRET was compared across identical acceptor-to-donor ratios, and measurements were restricted to regions of interest drawn at the PM. Cells with undetectable levels of E-FRET were assigned to a value of 0.

### Confocal microscopy

HEK293H cells expressing various ORAI1-YFP/CFP mutants and CFP-CAD were imaged with 445-nm (CFP) and 515-nm (YFP) laser diodes. 100× images were taken with an Andor XDI Revolution spinning disk confocal microscope equipped with a 100× oil immersion objective. 40× images were collected on a Yokogawa CSU-W1 spinning disk confocal microscope with a Hamamatsu Flash 4 camera using a 40× oil immersion objective. All confocal images were analyzed using NIH Fiji (ImageJ) software (National Institutes of Health). Cells with signal lower than 5× background were considered as “low ORAI1–expressing cells.” The remaining cells (>5× background) were analyzed for ORAI1 expression patterns based on visual assessment classified into two categories: at or near the PM or intracellular.

### scRNA-seq

Single-cell transcriptomics were conducted on PBMCs of the patient with ORAI1 p.H134P/p.L194P mutation. PBMC were thawed and sorted for live cells with Annexin V (cat. no. 640912; BioLegend) in Annexin V binding buffer (cat. no. 422201; BioLegend). Live PBMCs were defined as Annexin V^–^ using an Aria II instrument (BD Biosciences) with a 70-μm nozzle. Live cells were collected in RPMI 1640 medium (cat. no. 10040CV; Corning) containing 20% FBS. Sorted live cells were stained using TotalSEQ Type A hashing antibodies (BioLegend; anti-human hashtag antibodies 1, 2, and 3) for patient demultiplexing and quantified using a Bio-Rad TC20 automated cell counter. Hashtag antibody–labeled cells were encapsulated into emulsion droplets using the 10x Genomics platform, and scRNA-seq libraries were constructed using Chromium Single Cell 3′ v3.1 Reagent Kit and 3′ Feature Barcode Kit (PN-120237 and PN-1000262) according to the manufacturer’s protocols. Batches were sequenced with 100-cycle run kits (26 bp Read1, 9 bp Index1, and 99 bp Read2) on NovaSeq 6000 Sequencing System (Illumina). Libraries were evaluated with an Agilent TapeStation 2200 using High-Sensitivity D1000 ScreenTape (Agilent Technologies).

Per-read per-library FASTQ files were generated from the BCL base call output of the Illumina platform using the bcl2fastq package (v2.20.0.422). FASTQ files were then processed using 10x Genomics Cell Ranger v7.0.0, specifically the “cellranger count” pipeline, to align reads to the hg19 human reference genome, to generate gene-barcode expression matrices, and to perform preliminary clustering and differential expression analysis (42). The CITE-seq-Count pipeline (v1.4.2, https://hoohm.github.io/CITE-seq-Count/) was used to count by unique molecular identifier (UMI) the Cell Hashing Oligo Tags in the raw sequencing reads of the Cell Hashing library FASTQ files and to build cell-hashtag count matrices using a whitelist of only the cell barcodes called as cells by Cell Ranger software from the gene expression analysis. Single-cell analyses were conducted using R Statistical Software (v4.3.2; R Core Team 2021) and the Seurat package (v5.0.1) (43). Visualization and figure generation were performed using ggplot2 (v3.4.4) (44).

### Quality control and batch correction

All samples underwent quality control and demultiplexing. Hashtag values were normalized using a centered log ratio and defined via Seurat’s HTOdemux function. After removing doublets, cells were filtered based on mitochondrial content (cells with >15% mitochondrial reads were excluded) and genetic coverage (cells with <1,000 UMI were excluded). Three infant HD control scRNA-seq samples were acquired from GSE168732: HD1 (1.7-year-old male), HD2 (3-year-old female), HD3 (5-year-old female). HD controls were reported to have no recent history of fever, infection, or immunization (45). Each HD sample was integrated within their dataset using Seurat’s native RPCAIntegration function. Gene expression levels for the HD samples were normalized on a per-cell basis using log normalization and then scaled by a factor of 10,000. The HD samples were filtered with the same quality threshold used for the ORAI1 patient sample. The HD dataset was then combined with data from the ORAI1 patient. A principal component analysis (PCA) was performed using 2,000 highly variable features over the first 50 components. As patient and controls were from independent experiments, batch correction was performed with the Harmony package (46). For the Harmony correction, the variables batch, patient sample, and mitochondrial percentage were regressed out, achieving convergence after 9 iterations. The corrected PCA output was used for all further analyses. A total of 15,728 cells passed the quality control filters and were used for analysis.

### Cell clustering and cell-type annotation

Using the batch-corrected PCA results, a Uniform Manifold and Approximation Projection (UMAP) and a t-distributed stochastic neighbor embedding plot were generated based on the first 50 dimensions and 20 nearest neighbors. Cell clusters were identified using the same settings as for the dimensional reduction plots, with a resolution parameter set at 0.7. To confirm cell-type identity, cells were grouped into broad PBMC types based on established marker genes: CD4^+^ T cells: *CD4*, *LEF1*, *MAL*, *TCF7*, *IL7R*, *CCR7*; CD8^+^ T cells: *CD8A*, *CD8B*, *TRGC2*, *CCL5*, *GZMH*; B cells: *CD19*, *MS4A1*, *CD79A*, *CD79B*, *PAX5*; NK cells: *NKG7*, *GNLY*, *NCR1*, *KLRD1*, *FCGR3A*; monocytes: *CD16*, *CD14*, *LYZ*, *S100A8*, *S100A9*, *FCN1* (Fig. S5 C). Within the T cell and NK cell populations, further subtypes were identified using the same clustering parameters mentioned above and annotated based on additional gene expression markers (Table S1).

### Differential gene expression and pathway analysis

To identify DEGs between the ORAI1-mutant patient and HD controls, each identified cell-type cluster was subdivided by the disease state. Due to unequal representation, myeloid lineage cell types were excluded from the differential expression analysis. Differential gene expression between the ORAI1-deficient patient and the controls was calculated using Seurat’s FindMarkers function. Genes were only considered significant if the calculated adjusted P value (P_adj_) was <0.05. The overlap of these dysregulated genes was assessed and plotted using the ComplexUpsetR package (47). Genes found to be significantly dysregulated in each cell type were used to identify the affected pathways. We used Qiagen’s Ingenuity Pathway Analysis (IPA) tool (https://digitalinsights.qiagen.com/IPA) to detect dysregulated pathways. IPA results were exported as tab-delimited files and then visualized as bar plots in R using the ggplot2 package. For visualization, we selected the top 20 dysregulated pathways with Z scores >1 or less than −1. Redundant or overlapping pathways were manually removed.

### NMF

To identify unique cell-type functions and their changes between the ORAI1-mutant patient and controls, we performed consensus nonnegative matrix factorization (cNMF) as described in reference (48). cNMF is based on the NMF implementation in scikit-learn v0.20.0. We isolated cells identified as either NK or T lymphocytes and converted the Seurat object to a scanpy object compatible with the cNMF program (49). We filtered out genes that were not expressed in at least 10 cells across all samples and removed any cells that had <500 genes. Harmony-corrected counts were used for further downstream analysis. We calculated the top 2,000 overdispersed genes to perform the NMF analysis. To determine the number of components (k) that would be most accurate, we calculated k over a range of 5–30, with each value having 200 iterations. We calculated the silhouette score and error as implemented in cNMF. We found k = 17 to be the smallest, most stable solution. The final consensus solution was determined by using a density threshold of 0.05 to exclude outlier solutions. We identified 9 GEPs with a mean score of >0.1 for at least one cell type. An additional three usages were sample-specific, and another five were not heavily utilized by any cell type or groups of cell types (average usage scores <0.02). Marker genes for each GEP were calculated using multiple least squares of regression of normalized z-scored gene expression against the consensus GEP usage matrix as implemented by cNMF. A functional enrichment test for differentially utilized GEP was subsequently performed using the top 100 marker genes per GEP using the EnrichR platform (50). cNMF analysis was performed in Python (v.3.7.0) using scanpy (v.1.6.0), pandas (v.1.1.3), numpy (v.1.19.2), matplotlib (v.3.3.2) (49, 51). After cNMF analysis, the cell × usage (K × G) matrix was superimposed onto the scanpy object with corresponding per cell values for each GEP per cell. This object was converted back into Seurat for subsequent analysis, quantification, and visualization.

### Statistical analyses

Experimental data analysis was performed using GraphPad Prism 10 software. The statistical significance of differences between experimental groups was determined by unpaired Student’s *t* test, one-way ANOVA, Wilcoxon’s rank sum test, and right-tailed Fisher’s exact test as indicated in the figure legends. The significance of DEGs was determined using the default Wilcoxon ranked sum test. Genes were considered significantly different if they were expressed in at least 10% of cells, exhibited an absolute log_2_ fold change of ≥0.1, and had Padj ≤ 0.01. Differences were considered significant for P values <0.05. The P values are indicated in figure legends. All results are shown as means ± SEM; ns, not significant, *P < 0.05, **P < 0.01, and ***P < 0.001.

### Online supplemental material

Fig. S1 shows CMV retinitis in the patient with *ORAI1* p.L194P and p.H134P mutations. Fig. S2 shows reduced ORAI1-L194P expression and retention in the cytosol. Fig. S3 shows defective SOCE in T cells of patients with ORAI1 and STIM1 mutations. Fig. S4 shows reduced cytokine production by CD4^+^ and CD8^+^ T cells of the ORAI1 mutant patient. Fig. S5 shows enhanced T cell activation in the ORAI1 mutant patient. Table S1 lists marker genes used for cell-type annotation by scRNA-seq.

## Supplementary Material

Table S1 shows marker genes used for cell-type annotation by scRNA-seq.

## Data Availability

HD control samples were acquired from the Gene Expression Omnibus (GEO), accession number GSE168732. Raw sequencing data from the ORAI1 patient have been deposited in the GEO under the accession number GSE299029.
