# Supplementary material for: ORAI1 mutation with mixed loss- and gain-of-function properties causes immunodeficiency and HLH
Source: J Hum Immun. 2025 Oct 30;1(4):e20250097. doi: 10.70962/jhi.20250097 (PMC12588105; doi:10.70962/jhi.20250097)
Supplement: Table S1 — shows marker genes used for cell-type annotation by scRNA-seq. [file jhi_20250097_tables1.docx]

**Supplementary Table 1.** Marker genes used for cell type annotation by scRNA-seq. Abbreviations: EM, effector memory; Double Pos., CD4^+^ CD8^+^ T cells

| **gene** | **p_val** | **Log_2_FC** | **pct.1** | **pct.2** | **p_adj** | **cluster** |
| --- | --- | --- | --- | --- | --- | --- |
| LINC02446 | 7.71E-120 | 3.96 | 0.70 | 0.06 | 1.31E-115 | Naive CD8 |
| CD8B | 5.99E-51 | 2.33 | 0.60 | 0.15 | 1.02E-46 | Naive CD8 |
| NELL2 | 6.38E-47 | 1.53 | 0.75 | 0.34 | 1.09E-42 | Naive CD8 |
| S100B | 1.33E-42 | 3.97 | 0.29 | 0.02 | 2.26E-38 | Naive CD8 |
| KLRK1 | 3.35E-15 | 1.32 | 0.45 | 0.21 | 5.70E-11 | Naive CD8 |
| NUCB2 | 2.64E-12 | 1.01 | 0.46 | 0.27 | 4.50E-08 | Naive CD8 |
| CARS | 3.03E-11 | 1.92 | 0.22 | 0.07 | 5.15E-07 | Naive CD8 |
| ACTN1 | 5.48E-10 | 1.86 | 0.21 | 0.07 | 9.33E-06 | Naive CD8 |
| YBX3 | 5.92E-10 | 1.53 | 0.21 | 0.09 | 1.01E-05 | Naive CD8 |
| AIF1 | 6.12E-10 | 1.40 | 0.31 | 0.13 | 1.04E-05 | Naive CD8 |
| ARMH1 | 2.19E-09 | 1.49 | 0.27 | 0.11 | 3.72E-05 | Naive CD8 |
| RUNX2 | 4.40E-09 | 1.50 | 0.20 | 0.09 | 7.49E-05 | Naive CD8 |
| SNED1 | 8.34E-09 | 1.29 | 0.23 | 0.12 | 1.42E-04 | Naive CD8 |
| KCNQ5 | 1.56E-08 | 1.17 | 0.30 | 0.16 | 2.65E-04 | Naive CD8 |
| TRABD2A | 1.81E-08 | 1.06 | 0.35 | 0.20 | 3.08E-04 | Naive CD8 |
| APBA2 | 5.83E-08 | 1.11 | 0.34 | 0.18 | 9.93E-04 | Naive CD8 |
| TIMP1 | 1.37E-39 | 1.09 | 0.33 | 0.15 | 2.33E-35 | CD4 EM |
| EPHA4 | 2.53E-30 | 1.30 | 0.24 | 0.09 | 4.30E-26 | CD4 EM |
| TRADD | 8.77E-29 | 1.05 | 0.30 | 0.14 | 1.49E-24 | CD4 EM |
| AQP3 | 3.55E-25 | 1.26 | 0.21 | 0.09 | 6.04E-21 | CD4 EM |
| PASK | 6.90E-23 | 1.18 | 0.22 | 0.09 | 1.18E-18 | CD4 EM |
| FHIT | 2.06E-98 | 1.21 | 0.63 | 0.36 | 3.51E-94 | Naive CD4 |
| LEF1 | 5.76E-79 | 1.08 | 0.58 | 0.35 | 9.81E-75 | Naive CD4 |
| MAL | 3.12E-43 | 1.03 | 0.44 | 0.26 | 5.31E-39 | Naive CD4 |
| CCR7 | 5.43E-43 | 1.25 | 0.33 | 0.18 | 9.25E-39 | Naive CD4 |
| AC139720.1 | 4.79E-26 | 1.18 | 0.21 | 0.12 | 8.16E-22 | Naive CD4 |
| TRABD2A | 1.00E-25 | 1.03 | 0.31 | 0.18 | 1.71E-21 | Naive CD4 |
| IGF1R | 2.12E-22 | 1.07 | 0.22 | 0.13 | 3.62E-18 | Naive CD4 |
| ITGA6 | 7.77E-20 | 1.12 | 0.22 | 0.12 | 1.32E-15 | Naive CD4 |
| GIMAP2 | 1.38E-19 | 1.15 | 0.21 | 0.12 | 2.36E-15 | Naive CD4 |
| LINC01550 | 3.95E-18 | 1.02 | 0.20 | 0.12 | 6.73E-14 | Naive CD4 |
| AIF1 | 2.52E-17 | 1.10 | 0.22 | 0.12 | 4.29E-13 | Naive CD4 |
| GIMAP5 | 3.47E-16 | 1.04 | 0.21 | 0.12 | 5.91E-12 | Naive CD4 |
| RETREG1 | 2.34E-15 | 1.07 | 0.21 | 0.11 | 3.99E-11 | Naive CD4 |
| PHF21A | 6.87E-13 | 1.05 | 0.30 | 0.20 | 1.17E-08 | Tox Pos. T Cells |
| TOX | 2.14E-11 | 1.31 | 0.34 | 0.17 | 3.64E-07 | Tox Pos. T Cells |
| MED13 | 1.25E-10 | 1.02 | 0.32 | 0.19 | 2.13E-06 | Tox Pos. T Cells |
| EP400 | 3.85E-10 | 1.33 | 0.24 | 0.12 | 6.55E-06 | Tox Pos. T Cells |
| STK39 | 1.34E-09 | 1.01 | 0.28 | 0.19 | 2.28E-05 | Tox Pos. T Cells |
| UBR3 | 8.90E-09 | 1.15 | 0.28 | 0.15 | 1.52E-04 | Tox Pos. T Cells |
| CCL5 | 0.00E+00 | 1.76 | 0.96 | 0.34 | 0.00E+00 | CD8 EM |
| NKG7 | 0.00E+00 | 1.57 | 0.95 | 0.34 | 0.00E+00 | CD8 EM |
| GZMH | 1.35E-266 | 2.24 | 0.73 | 0.18 | 2.30E-262 | CD8 EM |
| CST7 | 2.90E-178 | 1.53 | 0.74 | 0.27 | 4.94E-174 | CD8 EM |
| ZEB2 | 6.03E-166 | 1.65 | 0.68 | 0.24 | 1.03E-161 | CD8 EM |
| GZMA | 1.64E-125 | 1.33 | 0.68 | 0.29 | 2.80E-121 | CD8 EM |
| CD8A | 3.63E-98 | 2.28 | 0.35 | 0.08 | 6.18E-94 | CD8 EM |
| TRGC2 | 2.16E-94 | 2.90 | 0.26 | 0.04 | 3.69E-90 | CD8 EM |
| CD8B | 2.20E-76 | 1.74 | 0.35 | 0.10 | 3.75E-72 | CD8 EM |
| AC243829.2 | 3.11E-70 | 2.54 | 0.23 | 0.05 | 5.30E-66 | CD8 EM |
| FGFBP2 | 1.07E-68 | 1.33 | 0.44 | 0.17 | 1.82E-64 | CD8 EM |
| EFHD2 | 3.97E-55 | 1.10 | 0.48 | 0.23 | 6.76E-51 | CD8 EM |
| LYAR | 6.46E-53 | 1.43 | 0.36 | 0.15 | 1.10E-48 | CD8 EM |
| HLA-DPB1 | 3.80E-47 | 1.06 | 0.46 | 0.23 | 6.48E-43 | CD8 EM |
| PLEK | 1.82E-43 | 1.44 | 0.31 | 0.12 | 3.10E-39 | CD8 EM |
| C12orf75 | 1.91E-42 | 1.19 | 0.38 | 0.18 | 3.25E-38 | CD8 EM |
| GZMK | 3.13E-40 | 1.62 | 0.24 | 0.09 | 5.33E-36 | CD8 EM |
| C1orf21 | 4.67E-37 | 1.48 | 0.25 | 0.09 | 7.96E-33 | CD8 EM |
| TGFBR3 | 7.07E-32 | 1.04 | 0.36 | 0.19 | 1.20E-27 | CD8 EM |
| CCL4 | 7.56E-30 | 1.22 | 0.25 | 0.11 | 1.29E-25 | CD8 EM |
| CMC1 | 2.33E-29 | 1.07 | 0.32 | 0.18 | 3.97E-25 | CD8 EM |
| KLRG1 | 6.43E-26 | 1.28 | 0.23 | 0.11 | 1.09E-21 | CD8 EM |
| APOBEC3G | 1.12E-25 | 1.17 | 0.25 | 0.12 | 1.91E-21 | CD8 EM |
| PPP2R2B | 3.53E-23 | 1.12 | 0.24 | 0.11 | 6.01E-19 | CD8 EM |
| TRG-AS1 | 7.97E-21 | 1.04 | 0.25 | 0.14 | 1.36E-16 | CD8 EM |
| TYROBP | 3.33E-277 | 3.41 | 0.69 | 0.07 | 5.67E-273 | NK Cell |
| GZMB | 1.36E-257 | 2.40 | 0.84 | 0.19 | 2.32E-253 | NK Cell |
| FCER1G | 8.18E-243 | 3.83 | 0.58 | 0.05 | 1.39E-238 | NK Cell |
| NKG7 | 4.78E-241 | 1.43 | 0.98 | 0.42 | 8.14E-237 | NK Cell |
| KLRF1 | 4.80E-189 | 3.39 | 0.52 | 0.05 | 8.18E-185 | NK Cell |
| SPON2 | 1.81E-185 | 3.06 | 0.57 | 0.08 | 3.09E-181 | NK Cell |
| PRF1 | 4.21E-181 | 2.51 | 0.65 | 0.13 | 7.17E-177 | NK Cell |
| GZMA | 5.56E-170 | 1.59 | 0.85 | 0.30 | 9.46E-166 | NK Cell |
| CLIC3 | 2.41E-153 | 3.08 | 0.49 | 0.07 | 4.11E-149 | NK Cell |
| FGFBP2 | 3.19E-151 | 2.11 | 0.66 | 0.17 | 5.42E-147 | NK Cell |
| CST7 | 1.10E-145 | 1.49 | 0.82 | 0.32 | 1.87E-141 | NK Cell |
| FCGR3A | 8.33E-135 | 3.37 | 0.38 | 0.04 | 1.42E-130 | NK Cell |
| TRDC | 9.60E-131 | 3.25 | 0.39 | 0.04 | 1.64E-126 | NK Cell |
| IGFBP7 | 1.07E-128 | 5.16 | 0.27 | 0.01 | 1.83E-124 | NK Cell |
| KLRD1 | 3.05E-123 | 2.06 | 0.57 | 0.14 | 5.20E-119 | NK Cell |
| EFHD2 | 5.05E-121 | 1.63 | 0.69 | 0.23 | 8.61E-117 | NK Cell |
| KLRB1 | 2.77E-114 | 1.42 | 0.75 | 0.29 | 4.72E-110 | NK Cell |
| CTSW | 2.26E-107 | 1.44 | 0.72 | 0.28 | 3.86E-103 | NK Cell |
| LINGO2 | 6.06E-107 | 4.11 | 0.27 | 0.02 | 1.03E-102 | NK Cell |
| MCTP2 | 2.09E-99 | 1.85 | 0.54 | 0.15 | 3.55E-95 | NK Cell |
| AOAH | 5.75E-96 | 1.47 | 0.68 | 0.27 | 9.79E-92 | NK Cell |
| LYN | 8.73E-96 | 2.37 | 0.39 | 0.07 | 1.49E-91 | NK Cell |
| CCL3 | 2.37E-92 | 3.83 | 0.26 | 0.02 | 4.04E-88 | NK Cell |
| GNLY | 8.15E-62 | 1.77 | 1.00 | 0.38 | 1.39E-57 | CD56 Bright NK Cells |
| PPP1R9A | 5.83E-56 | 5.34 | 0.46 | 0.01 | 9.93E-52 | CD56 Bright NK Cells |
| NCAM1 | 1.53E-51 | 3.94 | 0.56 | 0.04 | 2.60E-47 | CD56 Bright NK Cells |
| XCL1 | 3.80E-40 | 5.28 | 0.34 | 0.01 | 6.47E-36 | CD56 Bright NK Cells |
| ZMAT4 | 6.41E-40 | 5.48 | 0.33 | 0.01 | 1.09E-35 | CD56 Bright NK Cells |
| ATP8B4 | 1.19E-39 | 3.86 | 0.46 | 0.03 | 2.02E-35 | CD56 Bright NK Cells |
| IL12RB2 | 3.78E-36 | 3.37 | 0.49 | 0.05 | 6.43E-32 | CD56 Bright NK Cells |
| TYROBP | 1.29E-31 | 2.11 | 0.67 | 0.15 | 2.20E-27 | CD56 Bright NK Cells |
| CMC1 | 1.96E-31 | 1.77 | 0.75 | 0.20 | 3.35E-27 | CD56 Bright NK Cells |
| XCL2 | 2.48E-30 | 3.53 | 0.38 | 0.03 | 4.22E-26 | CD56 Bright NK Cells |
| MAP3K8 | 1.82E-29 | 2.07 | 0.66 | 0.16 | 3.10E-25 | CD56 Bright NK Cells |
| KLRC1 | 4.72E-29 | 3.05 | 0.43 | 0.05 | 8.04E-25 | CD56 Bright NK Cells |
| KLRF1 | 2.90E-28 | 2.28 | 0.57 | 0.11 | 4.94E-24 | CD56 Bright NK Cells |
| FCER1G | 4.27E-27 | 2.18 | 0.57 | 0.12 | 7.28E-23 | CD56 Bright NK Cells |
| KLRD1 | 2.87E-25 | 1.74 | 0.67 | 0.20 | 4.89E-21 | CD56 Bright NK Cells |
| IL2RB | 1.65E-24 | 2.65 | 0.46 | 0.08 | 2.82E-20 | CD56 Bright NK Cells |
| GAB1 | 1.86E-23 | 4.95 | 0.21 | 0.01 | 3.17E-19 | CD56 Bright NK Cells |
| TRDC | 1.90E-23 | 2.45 | 0.47 | 0.09 | 3.24E-19 | CD56 Bright NK Cells |
| MAML3 | 1.58E-22 | 2.84 | 0.39 | 0.06 | 2.69E-18 | CD56 Bright NK Cells |
| GZMK | 1.57E-20 | 1.95 | 0.50 | 0.12 | 2.67E-16 | CD56 Bright NK Cells |
| AREG | 7.04E-20 | 2.09 | 0.51 | 0.13 | 1.20E-15 | CD56 Bright NK Cells |
| KLRC3 | 1.85E-18 | 2.94 | 0.30 | 0.04 | 3.16E-14 | CD56 Bright NK Cells |
| ITGAX | 3.52E-18 | 3.46 | 0.25 | 0.02 | 5.99E-14 | CD56 Bright NK Cells |
| IFITM3 | 3.75E-17 | 2.45 | 0.37 | 0.07 | 6.39E-13 | CD56 Bright NK Cells |
| DUSP2 | 1.51E-16 | 1.10 | 0.77 | 0.36 | 2.57E-12 | CD56 Bright NK Cells |
| MAFF | 6.25E-16 | 2.63 | 0.29 | 0.04 | 1.06E-11 | CD56 Bright NK Cells |
| MATK | 3.29E-15 | 1.76 | 0.43 | 0.11 | 5.60E-11 | CD56 Bright NK Cells |
| CTSW | 3.74E-15 | 1.16 | 0.74 | 0.34 | 6.36E-11 | CD56 Bright NK Cells |
| MCTP2 | 1.25E-14 | 1.43 | 0.56 | 0.20 | 2.12E-10 | CD56 Bright NK Cells |
| CD63 | 2.94E-14 | 1.18 | 0.64 | 0.27 | 5.01E-10 | CD56 Bright NK Cells |
| VAV3 | 1.26E-13 | 1.42 | 0.54 | 0.19 | 2.15E-09 | CD56 Bright NK Cells |
| HOPX | 3.46E-13 | 1.39 | 0.54 | 0.20 | 5.89E-09 | CD56 Bright NK Cells |
| GAS7 | 5.19E-13 | 2.85 | 0.25 | 0.04 | 8.84E-09 | CD56 Bright NK Cells |
| KLRC2 | 7.82E-13 | 2.97 | 0.23 | 0.03 | 1.33E-08 | CD56 Bright NK Cells |
| SAMD3 | 2.41E-12 | 1.23 | 0.59 | 0.25 | 4.10E-08 | CD56 Bright NK Cells |
| AOAH | 4.94E-12 | 1.00 | 0.67 | 0.32 | 8.42E-08 | CD56 Bright NK Cells |
| NCALD | 3.15E-11 | 1.24 | 0.57 | 0.25 | 5.37E-07 | CD56 Bright NK Cells |
| BST2 | 2.48E-10 | 1.14 | 0.58 | 0.27 | 4.22E-06 | CD56 Bright NK Cells |
| SKAP2 | 3.88E-10 | 2.02 | 0.25 | 0.05 | 6.61E-06 | CD56 Bright NK Cells |
| RIN3 | 5.26E-10 | 1.26 | 0.36 | 0.13 | 8.96E-06 | CD56 Bright NK Cells |
| TRGC1 | 1.05E-09 | 2.26 | 0.21 | 0.04 | 1.79E-05 | CD56 Bright NK Cells |
| PEAK1 | 4.46E-09 | 1.90 | 0.27 | 0.07 | 7.60E-05 | CD56 Bright NK Cells |
| HSH2D | 6.36E-09 | 1.69 | 0.29 | 0.08 | 1.08E-04 | CD56 Bright NK Cells |
| IFNG-AS1 | 1.13E-08 | 1.60 | 0.28 | 0.08 | 1.92E-04 | CD56 Bright NK Cells |
| TNFRSF18 | 1.19E-08 | 2.46 | 0.20 | 0.04 | 2.02E-04 | CD56 Bright NK Cells |
| KLRK1 | 1.42E-08 | 1.13 | 0.48 | 0.22 | 2.43E-04 | CD56 Bright NK Cells |
| PRF1 | 3.92E-08 | 1.03 | 0.46 | 0.20 | 6.68E-04 | CD56 Bright NK Cells |
| JARID2 | 4.27E-08 | 1.06 | 0.45 | 0.20 | 7.27E-04 | CD56 Bright NK Cells |
| PPBP | 2.15E-34 | 7.86 | 0.94 | 0.01 | 3.66E-30 | Double Pos. T Cells |
| NRGN | 3.89E-31 | 8.35 | 0.82 | 0.00 | 6.63E-27 | Double Pos. T Cells |
| GP1BB | 1.82E-28 | 8.05 | 0.82 | 0.00 | 3.10E-24 | Double Pos. T Cells |
| PF4 | 1.72E-22 | 8.07 | 0.65 | 0.00 | 2.93E-18 | Double Pos. T Cells |
| GNG11 | 1.67E-21 | 8.95 | 0.59 | 0.00 | 2.85E-17 | Double Pos. T Cells |
| CAVIN2 | 8.12E-21 | 8.39 | 0.59 | 0.00 | 1.38E-16 | Double Pos. T Cells |
| CMTM5 | 4.86E-18 | 9.97 | 0.47 | 0.00 | 8.28E-14 | Double Pos. T Cells |
| PRKAR2B | 3.15E-17 | 6.95 | 0.59 | 0.01 | 5.37E-13 | Double Pos. T Cells |
| RGS18 | 3.35E-16 | 7.41 | 0.53 | 0.00 | 5.71E-12 | Double Pos. T Cells |
| GP9 | 1.58E-14 | 10.70 | 0.35 | 0.00 | 2.69E-10 | Double Pos. T Cells |
| TUBB1 | 1.86E-14 | 7.56 | 0.47 | 0.00 | 3.17E-10 | Double Pos. T Cells |
| PTCRA | 2.41E-13 | 10.92 | 0.35 | 0.00 | 4.10E-09 | Double Pos. T Cells |
| F13A1 | 4.00E-13 | 7.93 | 0.41 | 0.00 | 6.82E-09 | Double Pos. T Cells |
| CLU | 1.15E-12 | 6.17 | 0.47 | 0.01 | 1.96E-08 | Double Pos. T Cells |
| HIST1H2AC | 7.24E-11 | 3.92 | 0.65 | 0.06 | 1.23E-06 | Double Pos. T Cells |
| MYL9 | 3.96E-10 | 8.24 | 0.29 | 0.00 | 6.74E-06 | Double Pos. T Cells |
| ARHGAP6 | 3.97E-10 | 8.49 | 0.29 | 0.00 | 6.77E-06 | Double Pos. T Cells |
| ABCC3 | 6.44E-09 | 8.66 | 0.24 | 0.00 | 1.10E-04 | Double Pos. T Cells |
| CA2 | 9.38E-09 | 8.82 | 0.24 | 0.00 | 1.60E-04 | Double Pos. T Cells |
| PTGS1 | 1.49E-08 | 8.65 | 0.24 | 0.00 | 2.54E-04 | Double Pos. T Cells |
| TSPAN33 | 4.01E-08 | 6.14 | 0.29 | 0.01 | 6.83E-04 | Double Pos. T Cells |
